# Identification of Flowering Regulatory Networks and Hub Genes Expressed in the Leaves of *Elymus sibiricus* L. Using Comparative Transcriptome Analysis

**DOI:** 10.3389/fpls.2022.877908

**Published:** 2022-05-16

**Authors:** Yuying Zheng, Na Wang, Zongyu Zhang, Wenhui Liu, Wengang Xie

**Affiliations:** ^1^The State Key Laboratory of Grassland Agro-ecosystems, Key Laboratory of Grassland Livestock Industry Innovation, Ministry of Agriculture and Rural Affairs, College of Pastoral Agriculture Science and Technology, Lanzhou University, Lanzhou, China; ^2^Key Laboratory of Superior Forage Germplasm in the Qinghai-Tibetan Plateau, Qinghai Academy of Animal Science and Veterinary Medicine, Xining, China

**Keywords:** flowering, *E. sibiricus*, transcriptome analysis, candidate genes, expression pattern

## Abstract

Flowering is a significant stage from vegetative growth to reproductive growth in higher plants, which impacts the biomass and seed yield. To reveal the flowering time variations and identify the flowering regulatory networks and hub genes in *Elymus sibiricus*, we measured the booting, heading, and flowering times of 66 *E. sibiricus* accessions. The booting, heading, and flowering times varied from 136 to 188, 142 to 194, and 148 to 201 days, respectively. The difference in flowering time between the earliest- and the last-flowering accessions was 53 days. Furthermore, transcriptome analyses were performed at the three developmental stages of six accessions with contrasting flowering times. A total of 3,526 differentially expressed genes (DEGs) were predicted and 72 candidate genes were identified, including transcription factors, known flowering genes, and plant hormone-related genes. Among them, four candidate genes (*LATE, GA2OX6, FAR3*, and *MFT1)* were significantly upregulated in late-flowering accessions. *LIMYB, PEX19, GWD3, BOR7, PMEI28, LRR*, and *AIRP2* were identified as hub genes in the turquoise and blue modules which were related to the development time of flowering by weighted gene co-expression network analysis (WGCNA). A single-nucleotide polymorphism (SNP) of *LIMYB* found by multiple sequence alignment may cause late flowering. The expression pattern of flowering candidate genes was verified in eight flowering promoters (*CRY, COL, FPF1, Hd3, GID1, FLK, VIN3*, and *FPA*) and four flowering suppressors (*CCA1, ELF3, Ghd7*, and *COL4*) under drought and salt stress by qRT-PCR. The results suggested that drought and salt stress activated the flowering regulation pathways to some extent. The findings of the present study lay a foundation for the functional verification of flowering genes and breeding of new varieties of early- and late-flowering *E. sibiricus*.

## Introduction

Over the past few decades, botanists have been studying the regulation of the timing of flowering (Kobayashi and Weigel, 2007). To reproduce successfully, plants must bloom at the right time (Zhao et al., 2020b). In the model plant *Arabidopsis thaliana*, several flowering regulatory pathways containing several genes have been reported for the molecular mechanism of flowering. The vernalization pathway regulates flowering through the occurrence of vernalization, mainly by repressing the expression of *FLOWERING LOCUS C* (*FLC*) (Srikanth and Schmid, 2011). The photoperiodic pathway regulates flowering through plants' sense of day and night length, and light signals are transformed into floral signals through *CONSTANS* (*CO*) (Andrés and Coupland, 2012). The gibberellin pathway is the pathway that induces flowering only through gibberellin signaling. It has been investigated in different plant species that both exogenous gibberellin and endogenous gibberellin can promote flowering (Osnato et al., 2012). The autonomic pathway is an endogenous regulator independent of other pathways and promotes flowering by suppressing the *FLC* gene expression, mainly involving *LUMINIDEPENDENS* (*LD*), *FLOWERING LOCUS D* (*FLD*), and *FLOWERING LOCUS KH DOMAIN* (*FLK*) (Michaels and Amasino, 2001). Some epigenetic factors are independent of photoperiod, gibberellin, and vernalization pathways called age pathways. The age pathway is mainly controlled by micro-RNA156 (miR156), and studies in *A. thaliana* have shown that nine miR156-targeted *SQUAMOSA PROMOTER BINDING protein-like* (*SPL*) genes are involved in flowering regulation (Zheng et al., 2019). In addition, stress-regulated flowering is an informal flowering regulatory pathway. Plants alter the flowering time to regulate their growth and developmental processes in response to changes in the external environmental conditions, such as drought and salt stress (Kazan and Lyons, 2016). In *A. thaliana*, integrator *FLOWERING LOCUS T* (*FT*), positive regulators *LEAFY* (*LFY*), and *SUPPRESSOR OF OVEREXPRESSION OF CO1* (*SOC1*) were induced to accelerate flowering and seed maturation under drought stress (Verslues and Juenger, 2011; Su et al., 2013). Photoperiod flowering time gene *GIGANTEA* (*GI*) is also a key regulator of drought escape response (Riboni et al., 2014). In wheat (*Triticum aestivum*) and barley (*Hordeum vulgare*), the effects of drought stress on flowering time are species-dependent. The genotypes with a winter major flowering time gene displayed a delay in flowering under drought; however,; the genotypes with a spring major flowering time gene performed a significant acceleration in flowering under drought (Gol et al., 2017). High-salt stress substantially represses the *FT* gene by a membrane-associated NAC transcription factor NTL8, resulting in the delay of flowering in *A. thaliana* (Kim et al., 2007). In addition, photoperiod flowering time gene *GI* has been reported to be degraded by the proteasome causing the delay of flowering in *A. thaliana* under salt stress (Kazan and Lyons, 2016).

Although, the flowering regulatory pathways are relatively conservative, different plants have their specificity with respect to flowering times. For instance, two specific pathways mediated by *Early heading date 1* (*Ehd1*) and *Days to heading on Chromosome 2* (*DTH2*), respectively, are found in rice (*Oryza sativa*) but not present in *Arabidopsis* (Naranjo et al., 2014). *Ehd1* under influencing by multigene promotes the expression of the florigen genes *Heading date 3a* (*Hd3a*) and *RICE FLOWERING LOCUS T1* (*RFT1)* in both short-day and long-day conditions (Kong et al., 2016). Similarly, *DTH2* expression induces the expression of *Hd3a* and *RFT1* to promote heading under long-day conditions (Zhao et al., 2015). Further studies are needed to explore whether these pathways only exist in *O. sativa* or co-exist in other Gramineae members. Therefore, there is a need of hour to reveal the flowering mechanism of different plants for breeding and production application.

*Elymus sibiricus* is a perennial, self-pollinating, and heterotetraploid (2n = 4x = 28) forage grass belonging to the genus *Elymus* of Triticeae in Poaceae (Xie et al., 2015b). It is widely distributed in high altitude areas in western and northern Eurasia; thus, it has good cold and drought resistance, making it suitable for ecological restoration of artificial grassland establishment in Qinghai-Tibet Plateau, China (Xie et al., 2015a). In *E. sibiricus*, the time of flowering determines whether it is suitable for hay or seed production. Late-flowering materials were selected for optimizing biomass yield, and early-flowering materials were selected for increasing seed yield production (Wolabu et al., 2016). The flowering process of herbage is affected by both external factors (light, temperature, and exogenous hormones) and internal factors (endogenous hormones, physiological conditions, and genetic characteristics), among which genetic characteristics are one of the most crucial factors (Shim and Jang, 2020). Exploring the complex process of flowering requires a high degree of coordination among various pathways. Until now, the genome of *E. sibiricus* has been sequenced but the data are not yet available (Xiong et al., 2021).

Hence, the transcriptomic study is a key approach on gene function and structure research of eukaryotic plants without reference genomes. Transcriptome sequencing is a technical method to study the transcription process of biological tissues or specific cells of a species at the RNA level under specific conditions (Van Dijk et al., 2018). It can obtain the gene expression information of species as a whole and create conditions for the connection between genome and proteome. As an effective tool, transcriptome sequencing is gradually applied to study herbage flowering. A previous report identified 77 flowering-related genes by transcriptome sequencing of inflorescences at different stages in orchardgrass (*Dactylis glomerata*) (Feng et al., 2017). In crested wheatgrass (*Agropyron cristatum*), a total of 113 flowering time-related genes, 123 MADS-box genes, and 22 *COL* genes were identified by RNA-seq of three successive growth stages (Zeng et al., 2017). Recently, weighted gene co-expression network analysis (WGCNA) has often been applied to transcriptome sequencing datasets. It is an analysis method based on multiple samples to explore the association between modules and focus phenotypes and mines the key genes in the co-expression network (Childs et al., 2011). This analysis method provides system-level insight into high sensitivity to genes and greatly reduces information loss (Pei et al., 2017).

Therefore, high-throughput transcriptome sequencing and WGCNA were used in this study to jointly discover the specific flowering hub genes and deduce flowering regulatory networks in the leaf of early- and late-flowering accessions in *E. sibiricus*. The expression patterns of flowering candidate genes were analyzed under abiotic stresses. This study could provide new insights to explore the actual flowering regulation mechanisms and help to cultivate high-yield and high-quality varieties of *E. sibiricus*.

## Materials and Methods

### Plant Materials and Growth Conditions

A total of 66 *E. sibiricus* accessions originated from four countries, including China, Russia, Mongolia, and Kyrgyzstan (Supplementary Table 1), were used in this study. A total of ten healthy, plump, and uniform seeds were selected from each accession and placed evenly in 9-cm Petri dishes covered with a filter paper. They were incubated and germinated in an incubator at a constant temperature 25°C with 16:8-h light/dark regime. When the seeds grew to 8 weeks old, three healthy seedlings were selected and transplanted to the Yuzhong test field of Lanzhou University, Gansu, China (latitude 35°34'N, longitude 103°34'E, elevation 1,720 m) with plant spacing of 0.5 m and row spacing of 0.4 m. We recorded the time of leaf sheath wrapping the spikelet inflated after flag leaf grown out as booting time, the time of three spikelets tip breaking through leaf sheath per plant as the heading time, and the time of the inferior palea and the palea opened with anther and filaments emerging as the flowering time. The number of days was calculated from 1st January according to the method provided by Xie et al. (2012). A number of five other inflorescences-related traits, such as reproductive branches per plant (RBP), spikelet number of rachis (SNR), floret number per spikelet (FNS), 100-seed weight (SW1), and breaking tensile strength (BTS), were also measured.

### RNA Extraction, Library Construction, and RNA-Seq

According to the records of booting, heading, and flowering time, three early-flowering accessions (PI598781, PI655199, and LQ10) and three late-flowering accessions (PI531665, and PI531669, PI595169) were chosen for the RNA-seq. The fresh leaves from each accession were collected in triplicates at the three developmental stages as follows: booting stage, heading stage, and flowering stage. The collected samples were stored at −80°C. The three sampling stages included booting from 15 May, heading from 21 May, and flowering from 27 May for early-flowering accessions, and booting from 5 June, heading from 10 June, and flowering from 21 June for late-flowering accessions.

Total RNAs were extracted from *E. sibiricus* samples using a UNIQ-10 Column TRIzol Total RNA Extraction Kit (Sangon Biotech, Shanghai). The purity, concentration, and integrity of RNA samples were detected by Nanodrop, Qubit 2.0, and Agilent 2100 methods, respectively. The qualified samples were used for library construction. A total of eighteen sequencing libraries were generated using Hieff NGS^®^ MaxUp II Dual-mode mRNA Library Prep Kit for Illumina^®^ MaxUp II (Yeasen Biotechnology, Shanghai). Library construction, RNA-seq, assembly, and quality analysis were conducted by Breeding Biotechnologies Company (Shaanxi, China) on Illumina HiSeq 2500 sequencing platform.

### Sequence Data Processing, *De novo* Assembly, and Annotation

Raw data were obtained after transcriptome sequencing of the library constructed by *E. sibiricus*. After filtering raw data, the joint sequences and primer sequences in the raw data were removed, empty sequences and low-quality sequences were screened out, and clean data were obtained for assembly. Unigenes were obtained by *de novo* transcriptome assembly by Trinity-v2.4.0.

The obtained functional gene sequences were annotated by Nr (NCBI non-redundant protein sequence), Gene Ontology (GO), Clusters of Orthologous Groups (COG), Karyotic Orthologous Groups (KOG), Kyoto Encyclopedia of Genes and Genomes (KEGG), and Swiss-Prot (annotated protein sequence) (Altschul et al., 1997). HMMER-v3.2.1 software was used for comparison with the Pfam (Protein Family) database (Eddy, 1998).

### Construction of Weighted Gene Co-Expression Networks and Identification of Modules

All DEGs were used for the construction of weighted gene co-expression networks. Filtering differentially expressed genes to remove low-expression genes, DEGs with FPKM > 0 in at least nine samples were retained. A total of 2,354 DEGs were obtained for WGCNA. The trait datasets mainly included the development time (DT), reproductive branches per plant (RBP), spikelet number of rachis (SNR), floret number per spikelet (FNS), 100-seed weight (SW1), and breaking tensile strength (BTS). The WGCNA assumed that the genetic network followed scale-free networks. The similarity matrix of gene co-expression and gene network formed the adjacency function, the similarity matrix was transformed into the adjacency matrix, and then, the expression correlation coefficient was calculated to construct the gene's hierarchical clustering tree. Modules were divided according to the clustering relationship between genes, and modules with similar expression patterns were merged. Finally, the correlation between phenotypic traits of modules and samples was analyzed, and the hub genes in the network were found by the R language package (Langfelder and Horvath, 2008).

### Validation Analysis of Transcriptome Data by qRT-PCR

The reliability of transcriptome data was verified by quantitative real-time PCR (qRT-PCR) experiments. The complementary DNA (cDNA) reverse transcription of total RNA was performed using PrimeScript^TM^ RT reagent Kit with gDNA Eraser (TaKaRa, Dalian, China). A total of twelve flowering-related candidate genes were selected randomly, and *Protein Phosphatase 2A* (*PP2A*) was used as a reference gene to evaluate the expression stability of the candidate genes (Zhang et al., 2019). The primers of a reference gene and candidate genes were designed by PrimerQuest Tool (http://sg.idtdna.com/Primerquest/Home/Index) (Supplementary Table 2).

The qRT-PCR was performed using an SYBR Premix Ex Taq^TM^ II kit (TaKaRa, Dalian, China) on a Roche LightCycler480 quantitative PCR instrument. The final reaction volume was 20 μl, and each reaction mixture contained abm^®^ EvaGreen qPCR MasterMix-no dye 10 μl, forward and reverse primers 0.8 μl, cDNA 1 μl, and ddH_2_O 7.4 μl. The following reaction procedures were used the enzyme activation at 95°C for 10 min, denaturation at 94°C for 15 s, annealing at 60°C for 1 min, denaturation and annealing for 40 cycles, and then the melting curve analyzed. The candidate gene expression level was calculated by the 2^−Δ*ΔCt*^ method.

### Gene Expression Analysis of Flowering Candidate Genes

In this study, the expression of flowering candidate genes in early- and late-flowering under different concentrations of salt stress and drought stress was investigated by a greenhouse pot experiment. The expression pattern of flowering candidate genes was studied by qRT-PCR technique. We combined qRT-PCR to study the expression patterns of candidate genes and elucidate the flowering molecular mechanism of *E. sibiricus*. Two accessions PI598781 and PI531665 were subjected to salt stress and drought stress at the heading stage. Under salt stress, 150, 200, and 250 mmol/L NaCl solutions (prepared with distilled water as mother liquor) were used to irrigate *E. sibiricus*. In the meanwhile, 10, 15, and 20% PEG6000 (polyethylene glycol) were used to irrigate *E. sibiricus* to simulate drought stress. After treatment for 6 h, the leaf samples were collected, stored at −80°C, and used to analyze the expression patterns of flowering candidate genes under different stress treatments. RNA extraction, cDNA preparation, and qRT-PCR analysis were carried out as presented above. Primer information on reference genes and candidate genes is shown in Supplementary Table 2.

## Results

### Flowering Time Variations of 66 *E. sibiricus* Accessions

**Figure 1:**
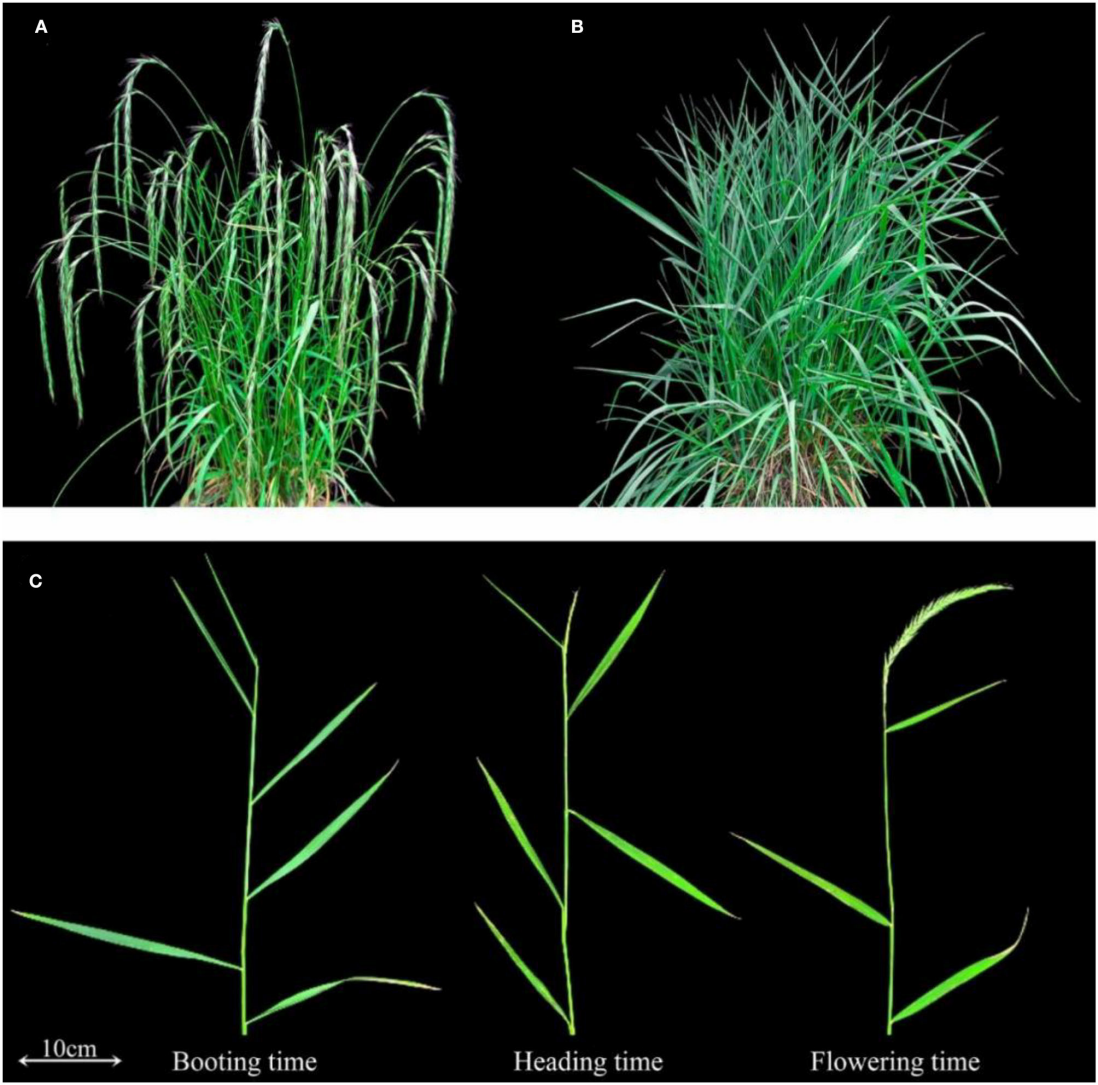
The image of *E. sibiricus* morphology. **(A)** Early-flowering accession. **(B)** Late-flowering accession. **(C)** The morphology of booting time, heading time, and flowering time in *E. sibiricus*. **(A)** and **(B)** were taken at 167 days according to the calculation method in this study.

**Figure 2:**
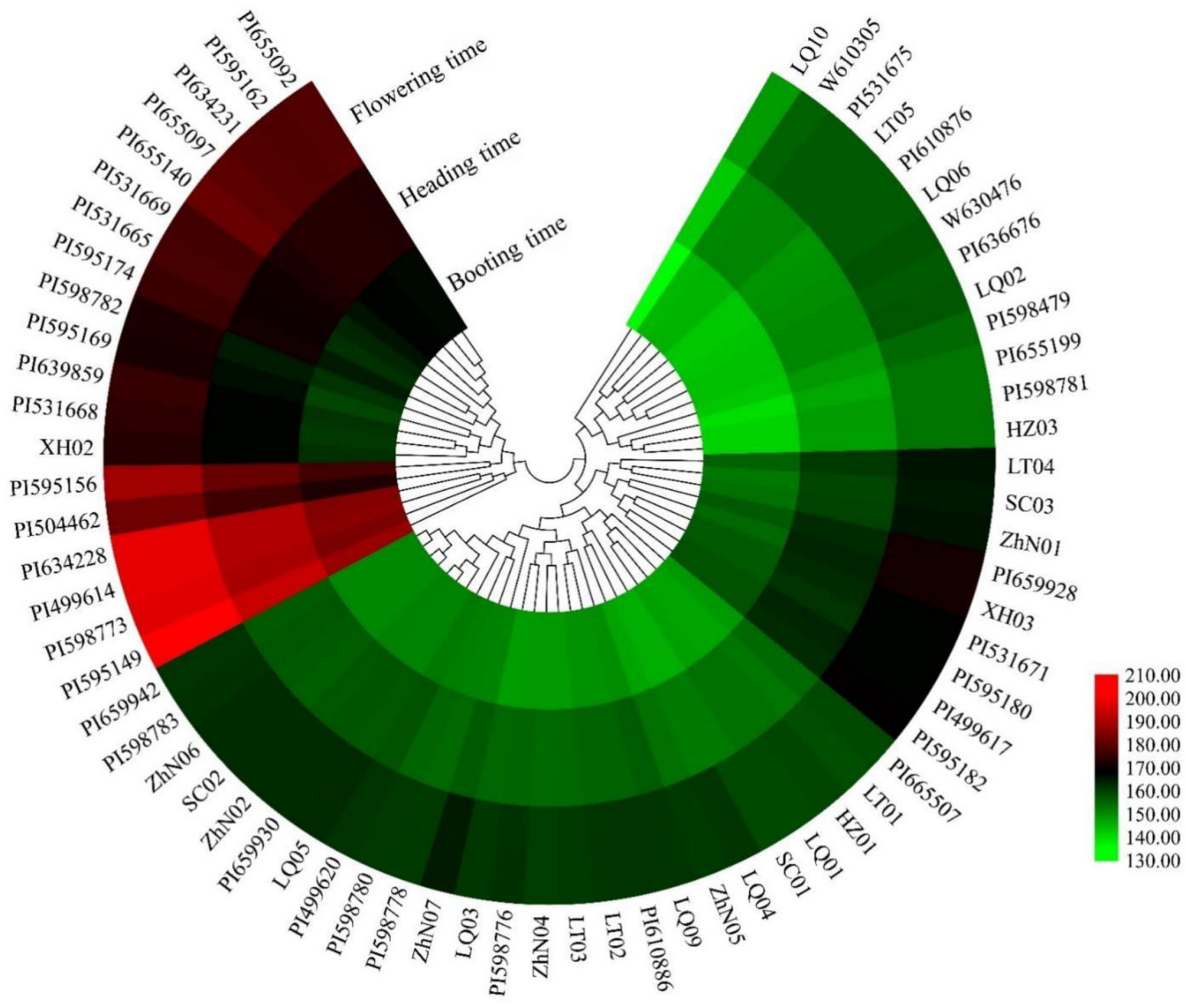
The heatmap of the booting time, heading time, and flowering time of 66 *E. sibiricus* accessions.

The booting, heading, and flowering times of 66 *E. sibiricus* accessions were measured (Figure 1). The booting time varied from 136 to 188 days, the heading time varied from 142 to 194 days, and flowering time varied from 148 to 201 days. The cluster heatmap showed that 66 *E. sibiricus* accessions were divided into two groups, one group included 43 early- and mid-flowering accessions and another group included 19 late-flowering accessions (Figure 2). The booting, heading, and flowering times for the earliest-flowering accession was 136, 142, and 148 days, respectively. For the last-flowering accession, the booting, heading, and flowering times were 188, 194, and 201 days, respectively. The difference in flowering times between the earliest-flowering accession and the last-flowering accession was 53 days. The variation coefficients of booting time, heading time, and flowering time across all accessions were 7.43, 6.98, and 6.92%, respectively. Hence, we selected three early-flowering accessions (PI598781, PI655189, and LQ10) and three late-flowering accessions (PI531665, PI531669, and PI595166) for further transcriptome analysis at booting, heading, and flowering stages (Table 1).

**Table 1:** The information of six *E. sibiricus* accessions for transcriptome sequencing.

**Sample name**	**Accession**	**Origin**	**Booting time (d)**	**Heading time (d)**	**Flowering time (d)**
E1	PI598781	Baikal, Buryat, Russia	141	148	153
E2	PI655199	Hongyuan, Sichuan, China	140	147	153
E3	LQ10	Luqu, Gansu, China	136	142	148
L1	PI531665	Beijing, China	162	170	177
L2	PI531669	Xining, Qinghai, China	163	170	177
L3	PI595169	Xinjiang, China	159	166	171

### Sequencing, Assembly, and Functional Annotation

A total of 18 samples (L1B, L1H, L1F, L2B, L2H, L2F, L3B, L3H, L3F, E1B, E1H, E1F, E2B, E2H, E2F, E3B, E3H, E3F) were employed to construct the cDNA library. After trimming, we got 119.63Gb databases (Supplementary Table 3). Pearson's correlation coefficient between the three biological replicates of different tissues varied from 0.69 to 0.98, indicating the high quality of the replicates (Supplementary Figure 1). *De novo* assembly of high-quality sequences through Trinity software produced more than 180,000 unigenes. The number of unigenes with the length of 200 to 300 bp was the highest (57,094). The number of unigenes decreased as the length of the sequence increased and increased again when the length of the sequence was >3,000 (Supplementary Figure 2). A total of 89.90% unigenes were successfully annotated in COG, GO, KEGG, KOG, Pfam, Swiss-Prot, TrEMBL, Nr, and Nt databases (Supplementary Table 4). Currently, homologous species matched results in Nr database showed that *Aegilops tauschii* (23,554, 29%) was the best match for *E. sibiricus*, followed by *H. vulgare* (15,885, 20%) (Supplementary Figure 3).

### Identification of Differentially Expressed Genes and KEGG Enrichment Analysis

**Figure 3:**
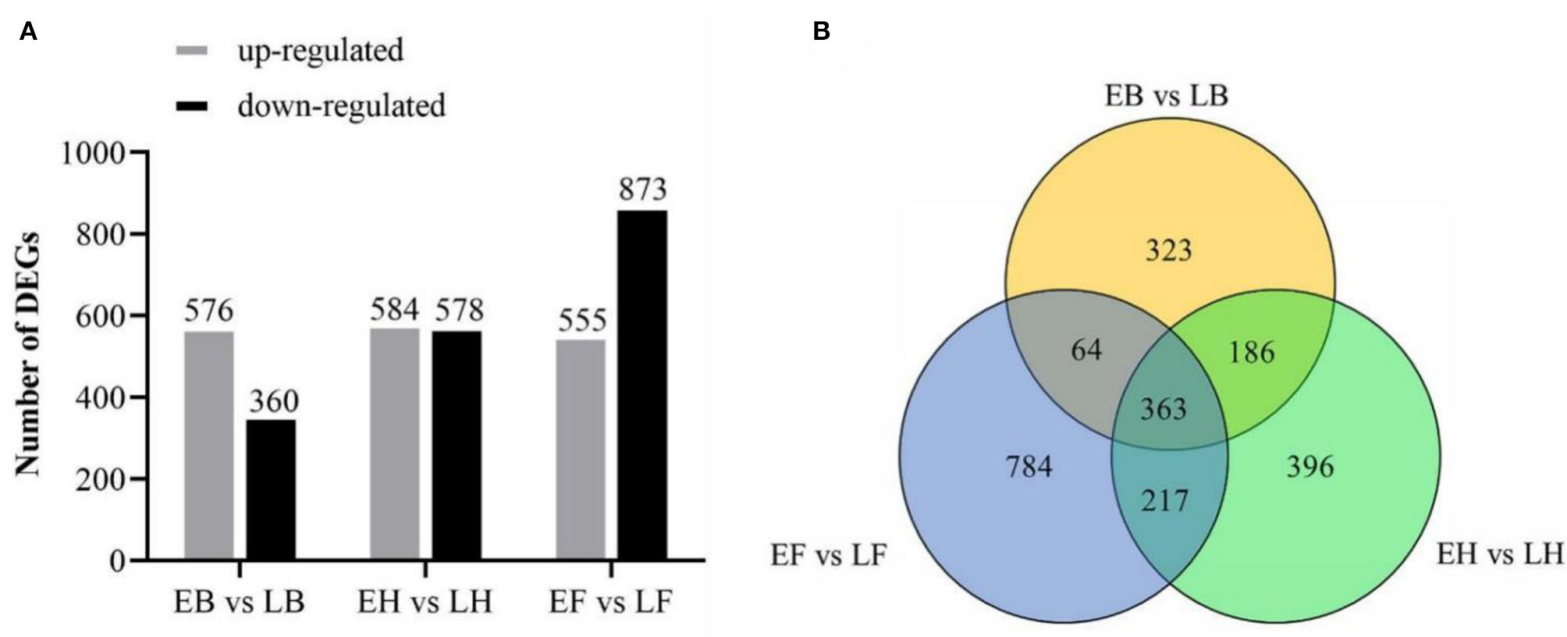
The number of DEGs of two opposing flowering accessions. **(A)** The column chart of DEGs number in three stages. **(B)** Venn diagram represents the number of overlapping DEGs.

The differentially expressed genes (DEGs) were filtered with expression levels FDR <0.01, log_2_-fold change ≥2. Based on this criterion, we identified 3,526 DEGs at booting, heading, and flowering stages, of which 936 (576 upregulated, 360 downregulated), 1,162 (584 upregulated, 578 downregulated), and 1,428 (555 upregulated, 873 downregulated) were predicted from “EB vs. LB,” “EH vs. LH,” “EF vs. LF,” respectively (Figure 3A). Among them, 363 DEGs were found to be common among three DEG sets. On the other hand, 323, 396, and 784 DEGs were found to be specific in “EB vs. LB,” “EH vs. LH,” and “EF vs. LF,” respectively (Figure 3B).

The biological functions and metabolic pathways in the three sets of DEGs were inquired based on the KEGG database. In EB vs. LB, 63 unigenes were annotated in 43 metabolic pathways, of which the “Glycolysis/Gluconeogenesis” was the most highly represented pathway. A total of 52 unigenes were annotated in 32 metabolic pathways in EH vs. LH. Among them, “flavonoid biosynthesis” was the most enriched pathway. In EF vs. LF, a total of 109 unigenes were enriched in 49 metabolic pathways, and the “starch and sucrose metabolism” was the highest enrich pathway, followed by the “galactose metabolism” pathway (Supplementary Figure 4).

**Figure 4:**
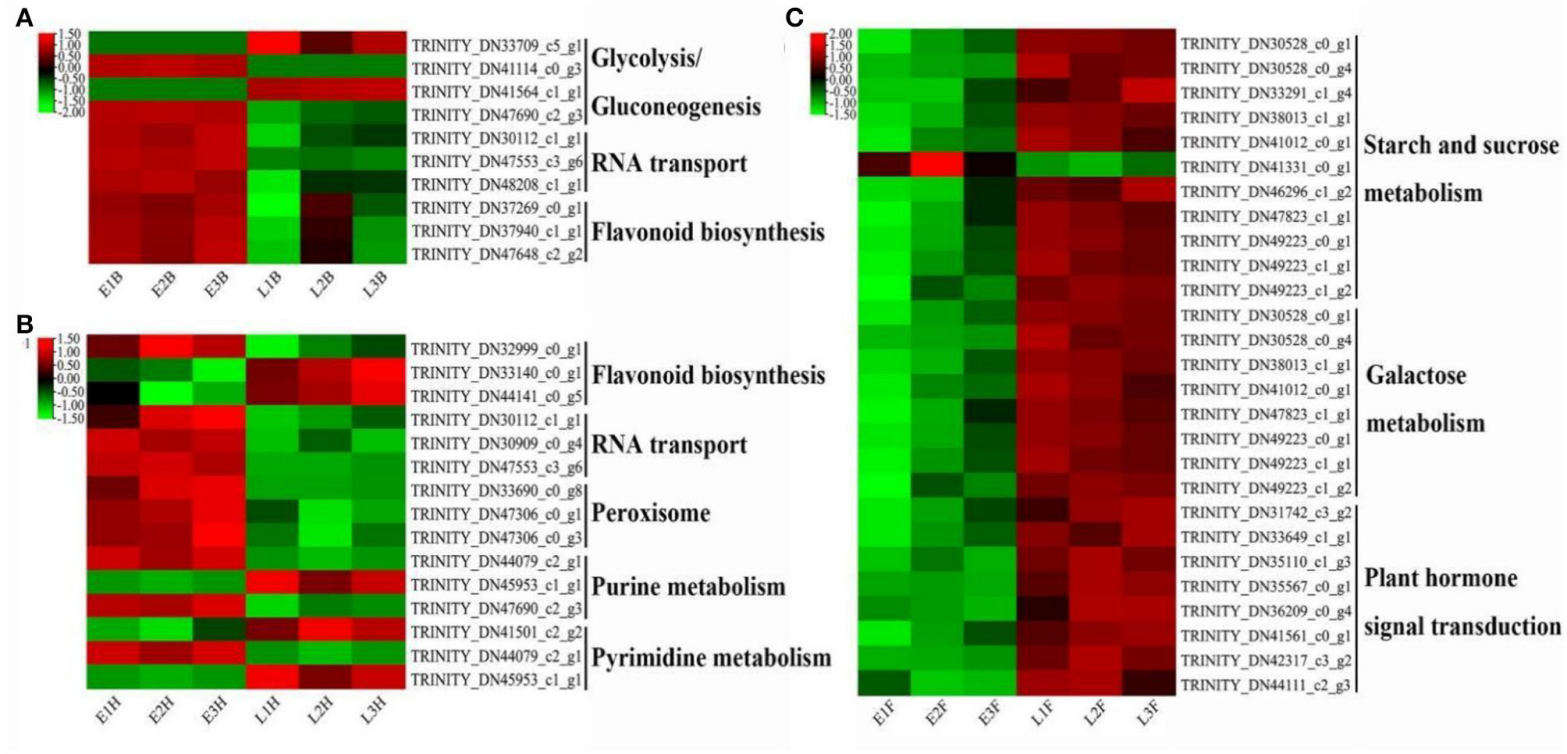
The heatmap of DEGs derived from KEGG pathways with two opposing flowering accessions. **(A)** EB vs. LB. **(B)** EH vs. LH. **(C)** EF vs. LF.

Lots of DEGs involved in glycolysis or gluconeogenesis, RNA transport, flavonoid biosynthesis, peroxisome, purine metabolism, pyrimidine metabolism, starch and sucrose metabolism, galactose metabolism, and plant hormone signal pathways were recognized as the candidate genes for flowering regulation. At the booting stage, DEGs involved in glycolysis or gluconeogenesis, RNA transport, and flavonoid biosynthesis were found to be highly expressed in the early-flowering accession, except for an alcohol dehydrogenase-like 3 gene (*TRINITY_DN33709_c5_g1*) and a glyceraldehyde-3-phosphate dehydrogenase gene *GAPCP1* (*TRINITY_DN41564_c1_g1*) (Figure 4A). At the heading stage, DEGs involved in RNA transport and peroxisome were upregulated in the early-flowering accession (Figure 4B). At the flowering stage, DEGs involved in starch and sucrose metabolism, galactose metabolic, and plant hormone signal pathways were highly expressed in late-flowering accessions relative to early-flowering accessions at the flowering stage, except for a glycosyl transferase family gene, *TRINITY_DN41331_c0_g1* (Figure 4C).

### Identification of Flowering Candidate Genes and Expression Patterns

**Figure 5:**
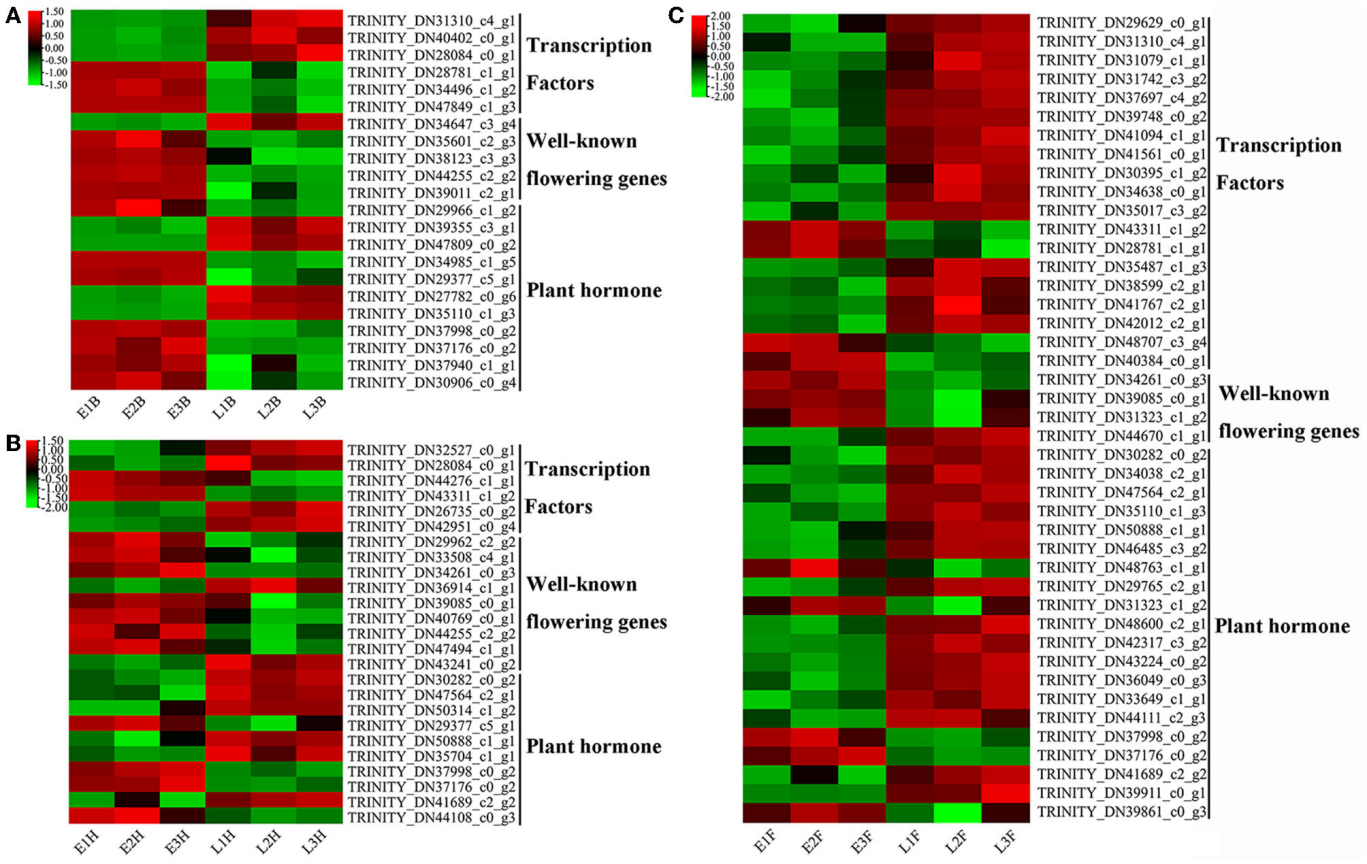
The heatmap of candidate genes expression in *E. sibiricus*. **(A)** EB vs. LB. **(B)** EH vs. LH. **(C)** EF vs. LF.

Based on the gene function annotation, 156 DEGs involved in the vernalization pathway (6), photoperiod pathway (82), circadian clock (12), gibberellin pathway (17), autonomic pathway (16), age pathway (5), and central integrator (18) were identified as flowering candidate genes (Supplementary Table 5). Among them, seventy-two candidate genes were divided into three parts, namely, transcription factors (27), known flowering genes (14), and plant hormone signal transduction (31). All transcription factors were identified from nine transcription factor families. The bZIP and MYB families have the most numbers of candidate genes, and the remaining 16 transcription factors were from the AP2/ERF (2), bHLH (3), Homebox (3), WRKY (2), HSF (2), MADS-box (1), and NAC family (3). Candidate genes identified in plant hormone signal transduction were involved in gibberellin, auxin, abscisic acid, jasmonic acid, and salicylic acid. A total of six genes involved in jasmonic acid, *TRINITY_DN39355_c3_g1* (Figure 5A), *TRINITY_DN47809_c0_g2* (Figure 5A), *TRINITY_DN50314_c1_g2* (Figure 5B), *TRINITY_DN34038_c2_g1* (Figure 5C), *TRINITY_DN30282_c0_g2* (Figures 5B,C), and *TRINITY_DN47564_c2_g1* (Figures 5B,C), were upregulated in late-flowering accessions; a total of two genes involved in salicylic acid, *TRINITY_DN30906_c0_g4* (Figure 5A), and *TRINITY_DN44108_c0_g3* (Figure 5B) were downregulated in late-flowering accessions. The homologous genes of known flowering genes were mostly upregulated in early-flowering accessions except for four genes. At the booting stage, a transcriptional repressor *LATE, TRINITY_DN34647_c3_g4*, was significantly upregulated in late-flowering accessions (Figure 5A). At the heading stage, two genes were significantly upregulated in late-flowering accessions which are gibberellin degradation enzyme gene *GA2OX6* (*TRINITY_DN43241_c0_g2*) and far-red elongated hypocotyl gene *FAR3* (*TRINITY_DN36914_c1_g1*) (Figure 5B). At the flowering stage, a flowering regulation repressor *MFT1, TRINITY_DN44670_c1_g1*, was significantly upregulated in late-flowering accessions (Figure 5C). These four candidate genes affected the formation of late-flowering accessions in *E. sibiricus*.

**Figure 6:**
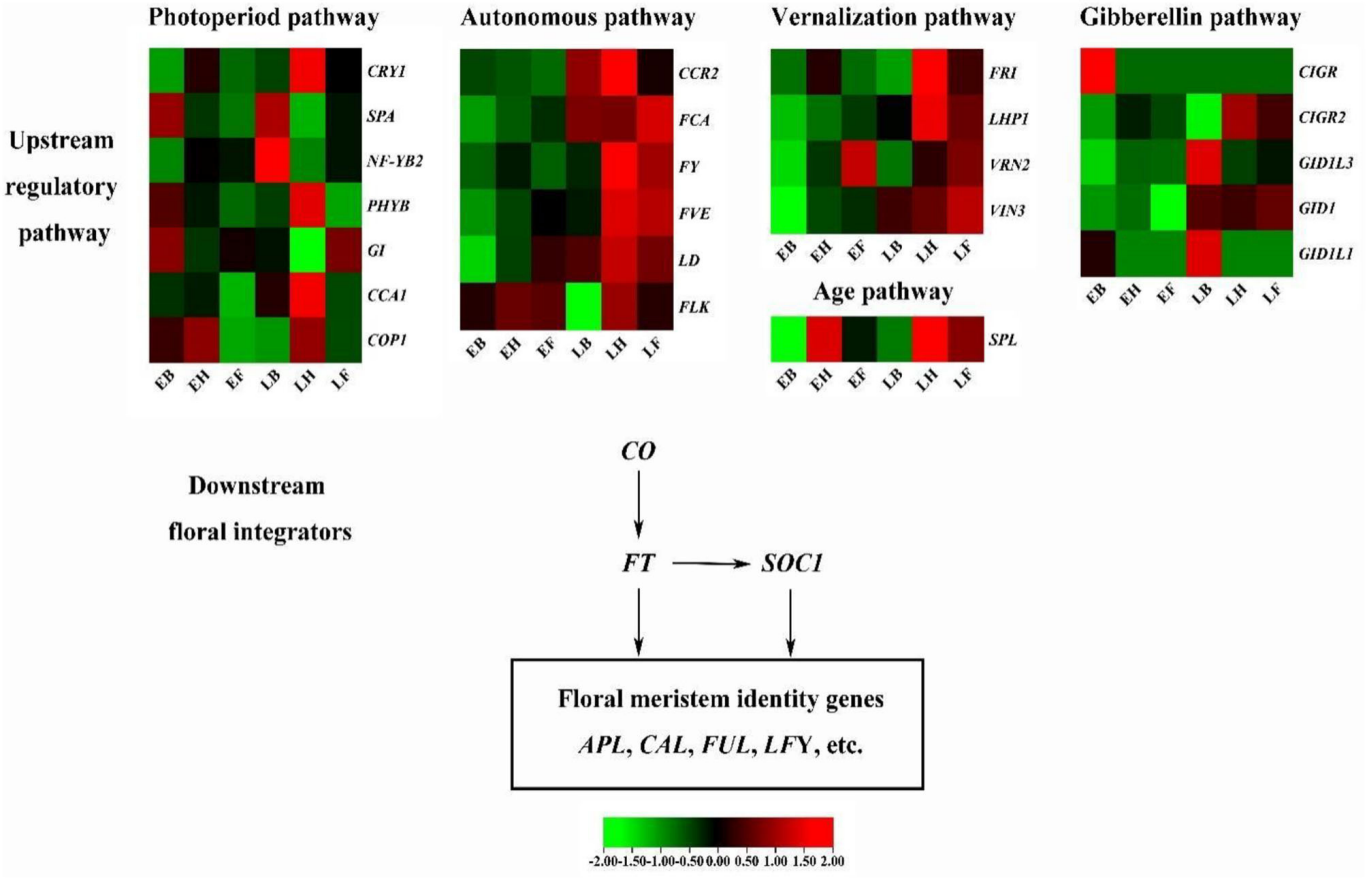
The putative flowering regulation network in *E. sibiricus*. Lines with arrows indicate activation.

Based on these candidate genes captured in multiple different flowering-related pathways and known regulatory networks in Arabidopsis and rice, we constructed a putative flowering regulation network in *E. sibiricus* (Figure 6).

### Construction of Weighted Gene Co-Expression Networks and Identification of Modules

**Figure 7:**
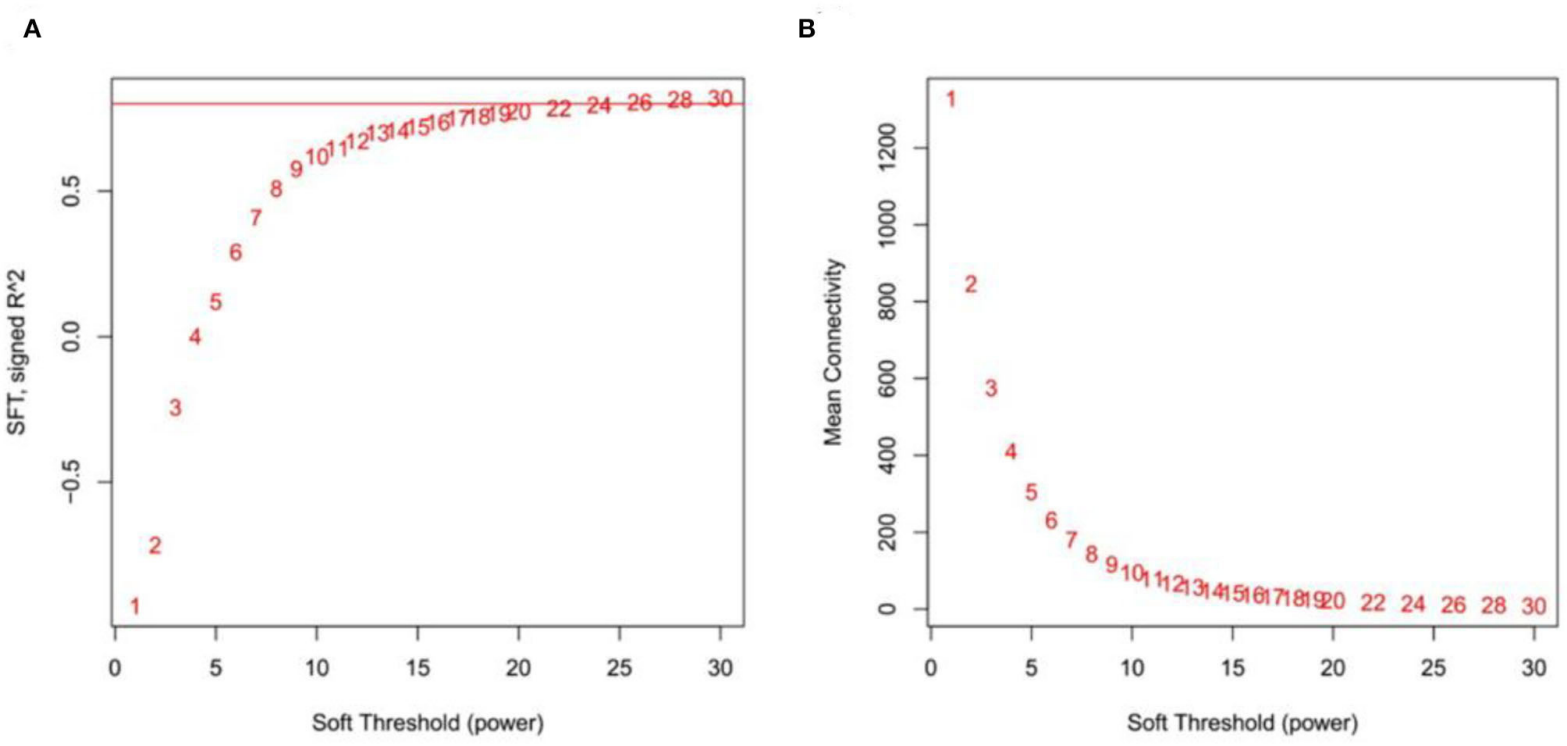
Determination soft threshold. **(A)** Scale independence. **(B)** Mean connectivity.

After screening the low expressed genes, 2,354 DEGs were retained for the WGCNA. A total of five distinct modules were identified *via* the block-wise module function, in which each module was labeled with different colors. Based on the approximate scale-free topology criterion, we have chosen the soft threshold power 24 when the correlation coefficient squared was 0.8 to define the adjacency matrix (Figure 7A). The corresponding relationship between the soft threshold and the mean value of gene linkage coefficient met the construction requirements (Figure 7B).

**Figure 8:**
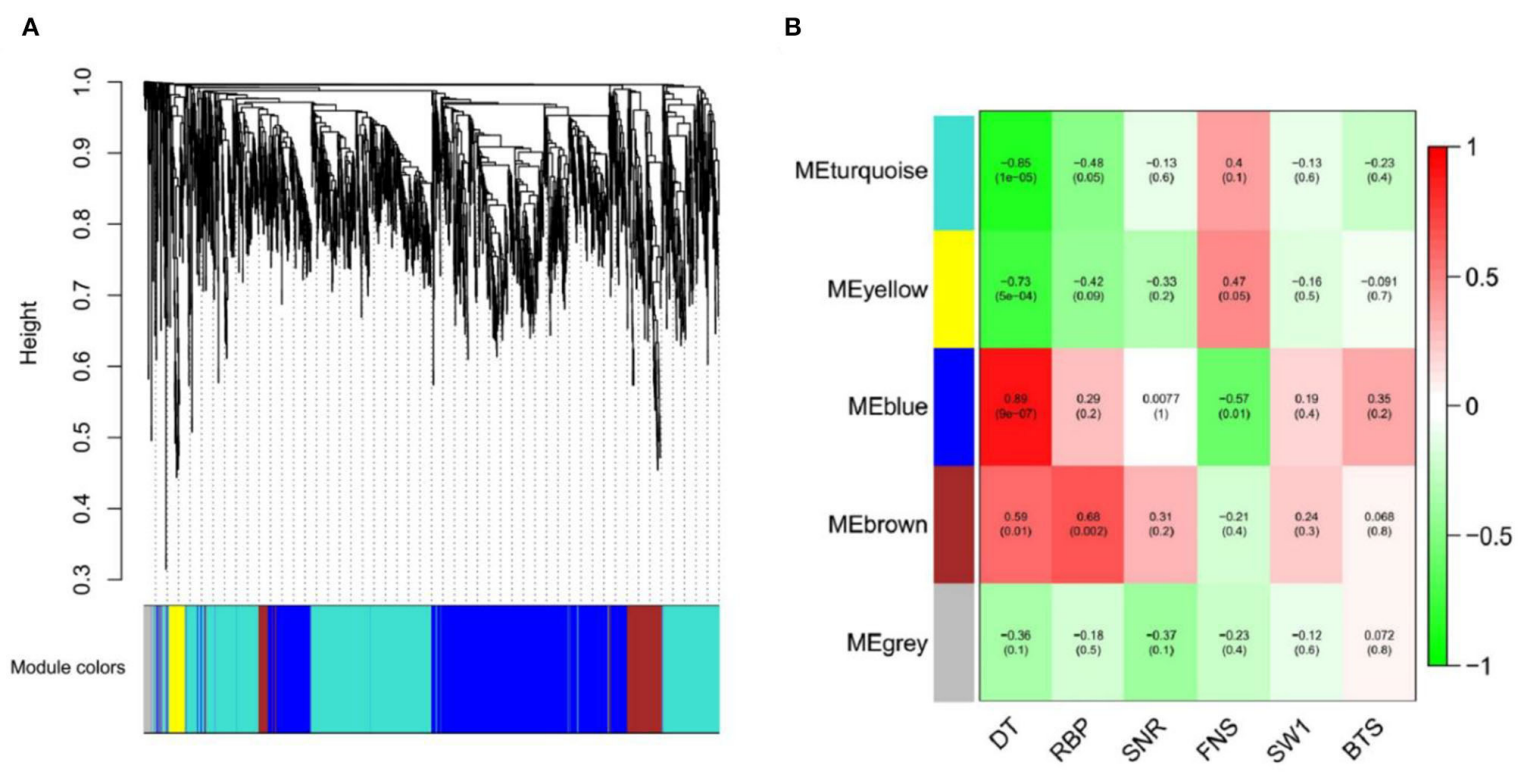
Gene cluster tree and module diagram, correlation heatmap between modules and traits. **(A)** Cluster Dendrogram. **(B)** Module–trait relationships.

According to the correlation coefficient between DEGs, a gene clustering tree was constructed, with modules of gray (60), turquoise (1,046), blue (982), brown (200), and yellow (66) (Figure 8A). Combined with Figure 8B, there was a significant difference among the five modules, especially the blue module (0.89), which was strongly related to the development time of flowering (DT); the turquoise module (−0.85) was negatively related to the DT trait. In addition, it could be seen that the blue and turquoise modules were still closely related to DT trait by the significance analysis between DT trait and DEGs (Supplementary Figure 5).

**Figure 9:**
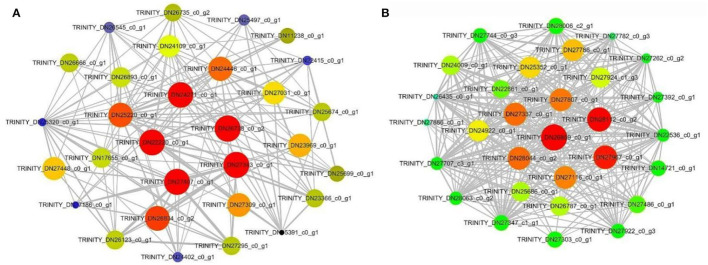
Blue module and turquoise module co-expression network visualization. **(A)** Blue. **(B)** Turquoise.

Therefore, we visualized the co-expression network between the blue module (Figure 9A) and the turquoise module (Figure 9B). A total of seven hub genes were identified in the two modules. The hub genes *L10-interacting MYB domain-containing protein* (*LIMYB*), *Peroxisome biogenesis protein 19* (*PEX19*), and *Glucan-water dikinase 3* (*GWD3*) were identified in the blue module; the hub genes *Boron transporter 7* (*BOR7*), *Pectin methylesterase inhibitor28* (*PMEI28*), *Leucine-rich repeat* (*LRR*), and E3 ubiquitin-protein ligase gene *AIRP2* were identified in the turquoise module (Table 2). The seven hub genes with high connectivity in the modules were more compatible with flowering development time, laying a foundation for the subsequent research on flowering genes of *E. sibiricus*.

**Table 2:** Hub genes.

**Number**	**Gene ID**	**Gene name**	**Module**	**Annotation**
1	TRINITY_DN26735_c0_g2	*LIMYB*	blue	MYB transcription factor
2	TRINITY_DN25320_c0_g1	*PEX19*	blue	Peroxisome biogenesis protein
3	TRINITY_DN26834_c0_g2	*GWD3*	blue	Pyruvate phosphate dikinase
4	TRINITY_DN27392_c0_g1	*BOR7*	turquoise	Boron transporter
5	TRINITY_DN27886_c0_g1	*PMEI28*	turquoise	Pectinesterase inhibitor
6	TRINITY_DN25352_c0_g1	*LRR*	turquoise	Leucine-rich repeat receptor-like protein kinase
7	TRINITY_DN27337_c0_g1	*AIRP2*	turquoise	E3 ubiquitin-protein ligase

### Allelic Variation Analysis of Hub Gene *LIMYB*

**Figure 10:**
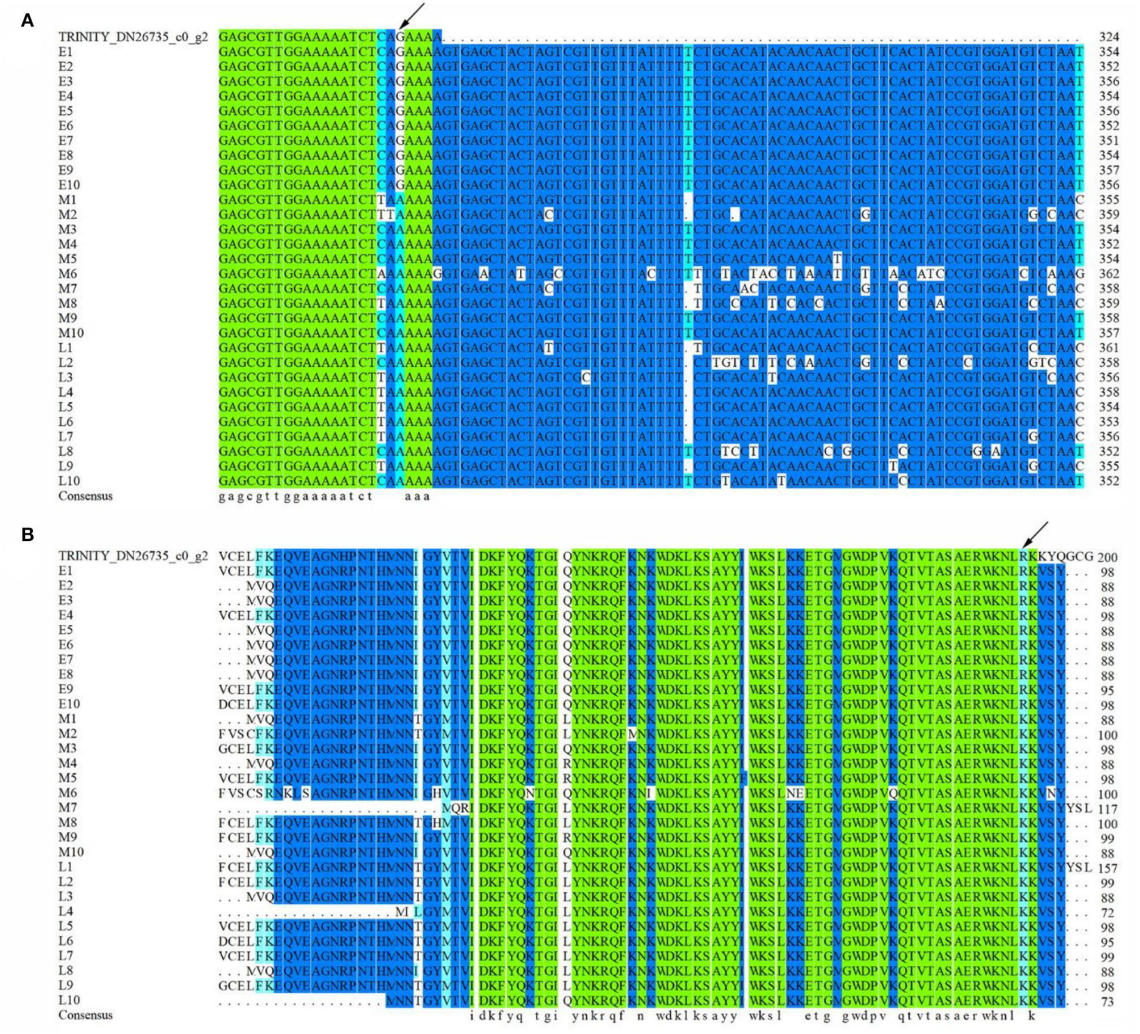
Multiple sequence alignment of hub gene *LIMYB*. **(A)** DNA sequences. **(B)** Amino acid sequences.

To reveal the potential allelic variation of hub gene *LIMYB* for flowering regulation, 30 *E. sibiricus* accessions were selected, including 10 early-flowering accessions, 10 mid-flowering accessions, and 10 late-flowering accessions, for allelic variation analysis. The result of multiple sequence alignment showed that a single-nucleotide polymorphism (SNP) was found in the 320-bp ORF region of the hub gene *LIMYB*. At 320 bp, 10 early-flowering accessions were completely consistent with the original sequence, whereas 10 mid-flowering accessions and 10 late-flowering accessions mutated from G (guanine) to A (adenine) (Figure 10A). The base mutation at 320 bp could have resulted in the amino acid mutation of the mid- and late-flowering accessions from R(arginine) to K(lysine) (Figure 10B).

### Validation of the Expression of Flowering-Related Genes

**Figure 11:**
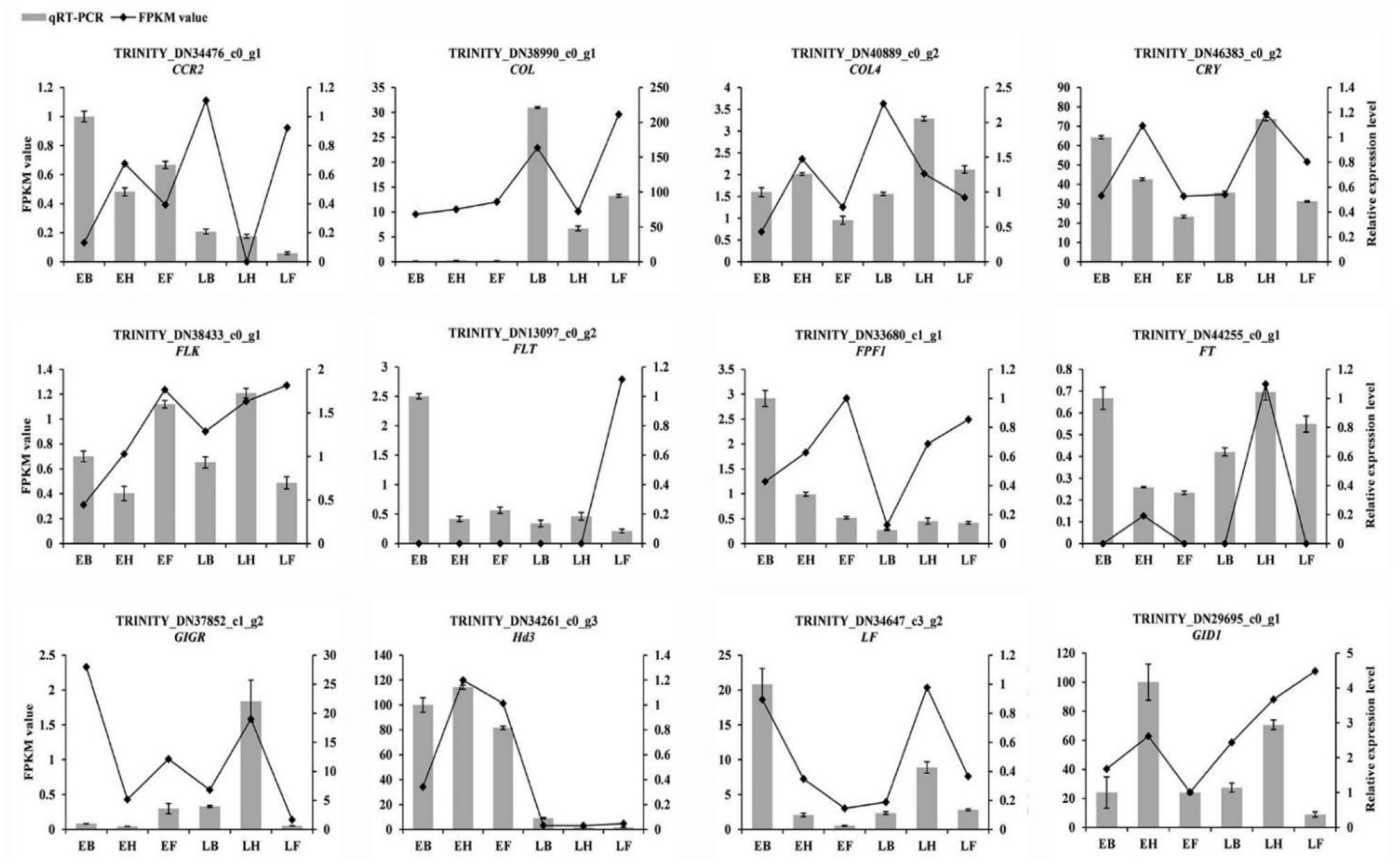
Validation of the expression by qRT-PCR. Bar charts indicate values of qRT-PCR. Line plots indicate values of FPKM value.

To confirm the authenticity of transcriptome sequencing data, twelve flowering candidate genes were selected for qRT-PCR verification, including *LF, CIGR, CCR2, CRY, COL, FPF1, FT, HD3, FLT, FLK, GID1*, and *COL4*. In E3 and L3 materials, *LF, CCR2, FPF1*, and *FLT* were all downregulated at heading and flowering time. The gene *COL* was upregulated in three stages, whereas *Hd3* was downregulated in three stages. The expression trends of the 12 flowering candidate genes in the early- and late-flowering accessions of *E. sibiricus* were similar to the transcriptome sequencing results (Figure 11). Linear regression analysis showed a positive correlation between the transcriptome sequencing data and the qRT-PCR results (*r* = 0.704, *p* < 0.05).

### Expression Analysis of Flowering-Related Genes Under Salt Stress

**Figure 12:**
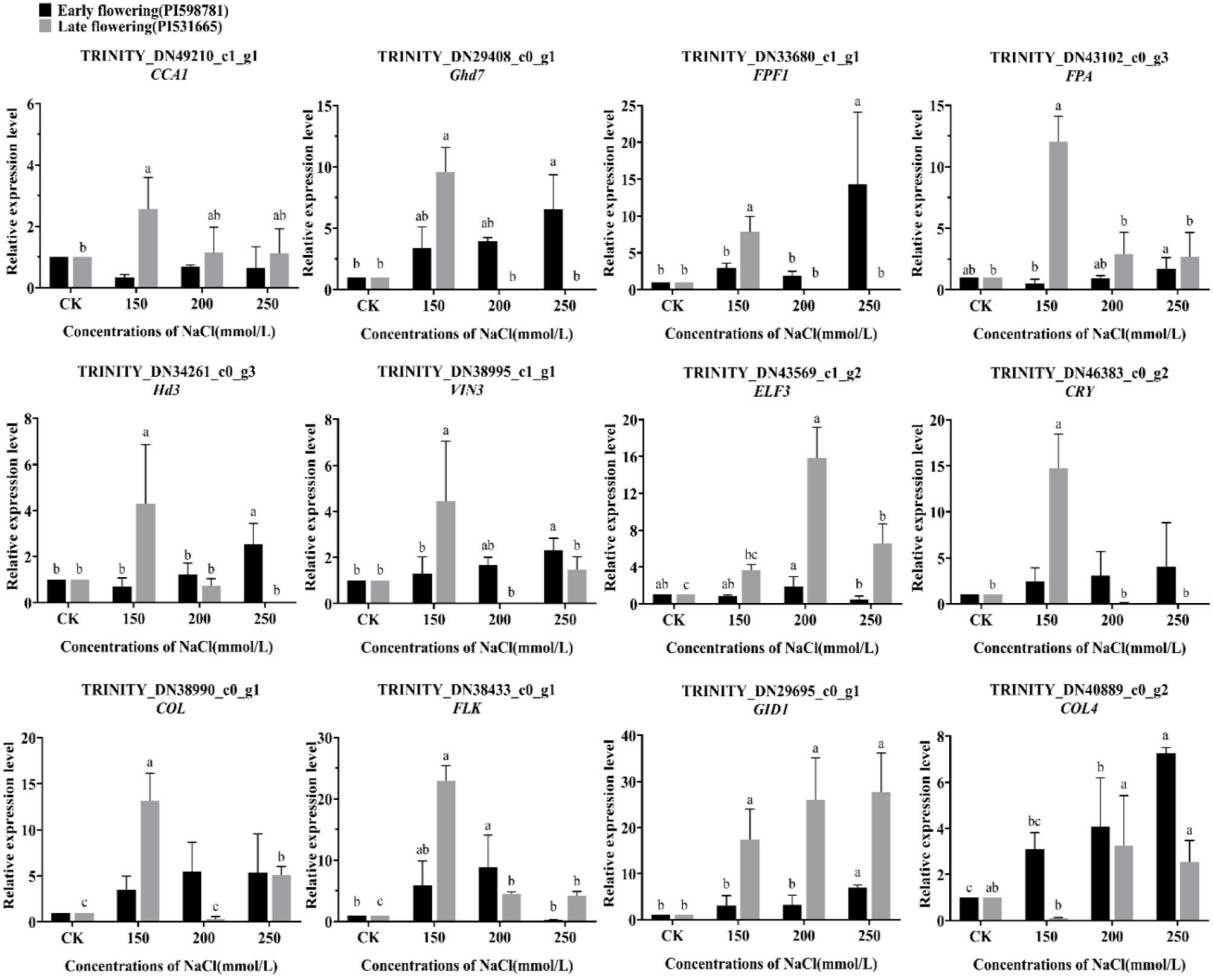
The expression pattern of 12 flowering candidate genes in the early- and late-flowering accessions under salt stress by qRT-PCR analysis.

A total of twelve flowering candidate genes, including *CCA1, Ghd7, FPF1, FPA, Hd3, VIN3, ELF3, CRY, COL, FLK, GID1*, and *COL4*, were selected for expression analysis of flowering-related genes under salt stress (Figure 12). In early-flowering accessions, the genes *Ghd7, FPF1, FPA, Hd3, VIN3, GID1*, and *COL4* were significantly upregulated under 150, 200, and 250 mmol/L NaCl treatments. The expression of genes *CCA1, ELF3, CRY*, and *COL* was not significant compared with the control under salt stress; however, the expression of genes *CRY* and *COL* was an upward trend under the three levels. Moreover, the gene *FLK* was only upregulated in 150 and 200 mmol/L NaCl treatments. In the late-flowering accessions, the genes *COL, FLK*, and *GID1* were significantly upregulated under 150, 200, and 250 mmol/L NaCl treatments. The *COL4* gene was the only one that was not significantly expressed under the three treatments. Most of the candidate genes, including *CCA1, Ghd7, FPF1, FPA, Hd3, VIN3*, and *CRY*, were just significantly upregulated under 150 mmol/L NaCl treatment, and their expression was downregulated when the salt stress increased. On the contrary, the expression of *ELF3* was significantly upregulated under 200 and 250 mmol/L NaCl treatments.

Under salt stress, the expression of most of these candidate genes was upregulated. The high concentration of salt stress stimulated the upregulation of candidate genes was confirmed in early-flowering accessions, whereas the high concentration of salt stress may block or suppress certain flowering pathways in late-flowering accessions.

### Expression Analysis of Flowering-Related Genes Under Drought Stress

**Figure 13:**
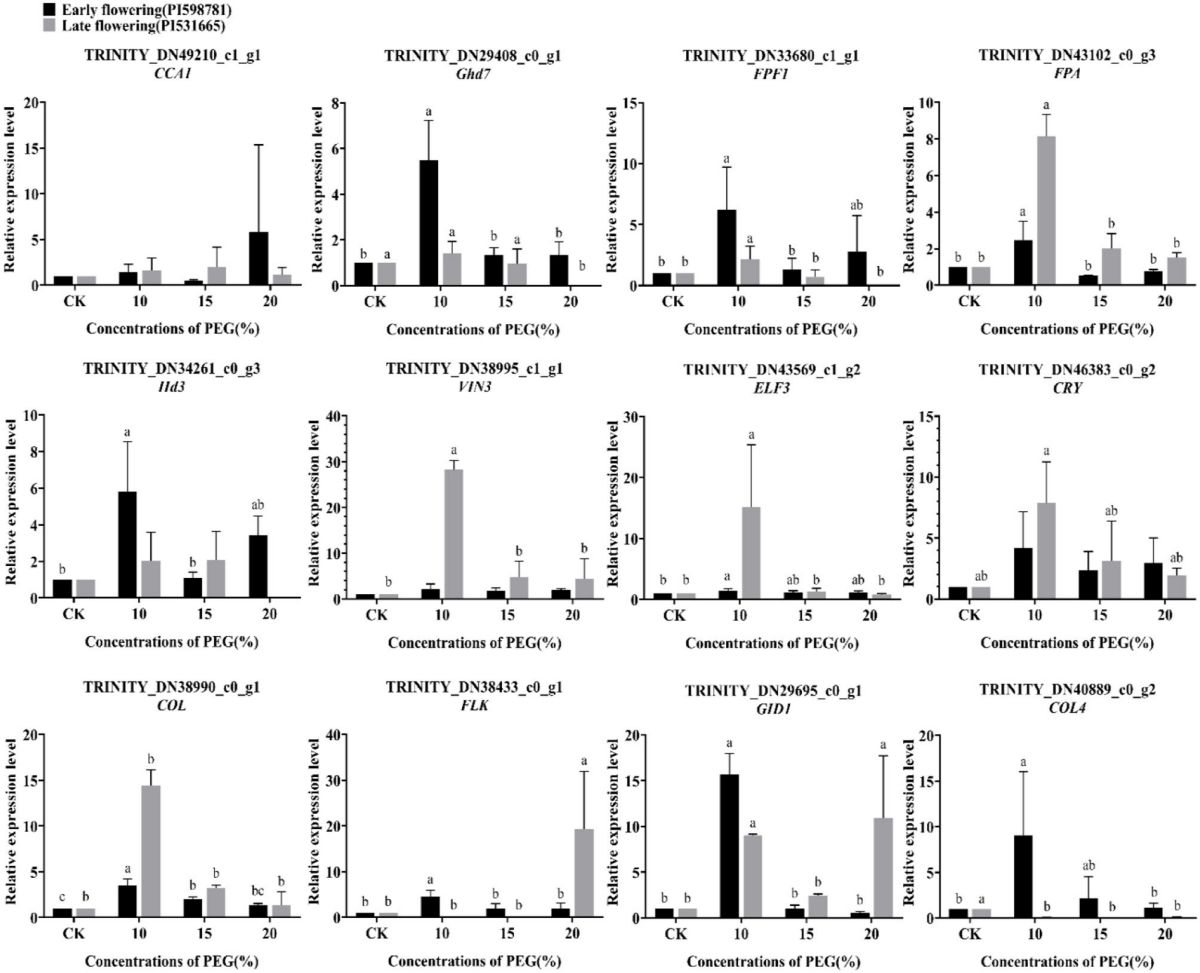
The expression pattern of 12 flowering candidate genes in the early- and late-flowering accessions under drought stress by qRT-PCR analysis.

Likewise, the expression analysis of twelve flowering candidate genes was detected under drought stress (Figure 13). In the early-flowering accessions, the expression of genes *Ghd7, FPF1, FPA, Hd3, ELF3, FLK, GID1, COL*, and *COL4* was significantly upregulated under 10% PEG6000 treatment. The expression quantities of these genes were low when the stress was strong, except that *COL* was also significantly upregulated under 15% PEG6000 treatment. The genes *CCA1, VIN3*, and *CRY* were not significantly expressed compared with the control. In the late-flowering accessions, the genes *FPF1, FPA, VIN3, ELF3, CRY*, and *GID1* were significantly upregulated under 10% PEG6000 treatment. Under 20% PEG6000 treatment, the flowering promoter *FLK* and the gibberellin receptor *GID1* were significantly upregulated, and the flowering suppressor *Ghd7* was downregulated. The result indicated that high-level stress-triggered changes in the expression of these genes led to drought escape. Interestingly, the gene *COL4* was downregulated under three levels of stress, suggesting that *COL4* may be only involved in early-flowering regulation. In addition, the genes *CCA1, Hd3*, and *COL* were not significantly expressed under drought stress.

A certain degree of drought stress could promote the response of the external environmental stimuli and endogenous signals to influence the flowering process. In the late-flowering accessions, the drought escape behavior of plants was more obvious.

## Discussion

### Variation of Flowering Time in *E. sibiricus*

In this study, the booting stage, heading stage, and flowering stage of 66 *E. sibiricus* accessions from different regions showed a great variation. These germplasms with different flowering times are beneficial for the breeding of early- and late-maturing varieties of *E. sibiricus*. Flowering is often affected by altitude, latitude, temperature, rainfall, and other factors (Linder, 2020). In general, high-altitude and high-latitude populations bloom earlier as compared to lower altitudes and latitudes. However, we found the correlation between the flowering time of 66 *E. sibiricus* accessions and altitude or latitude was weak. The weak correlation may be the result of environmental diversity in China and the different correlations between flowering time and environmental factors in different environments (Cremer et al., 1998; Huang et al., 2012). In future experiments, we can collect more information on environmental factors at the collection site to clarify the flowering mechanism of *E. sibiricus*.

### Multiple Gene Regulation Contributes to Flowering Variation in *E. sibiricus*

The flowering process of *E. sibiricus* is not controlled by a single gene, but by multiple flowering regulatory pathways. A large number of genes were differentially expressed in the flowering stage and heading stage between two opposite accessions which could contribute to the difference in flowering in *E. sibiricus*. In EH vs. LH, “flavonoid biosynthesis” was the most enriched pathway. *Dihydroflavonol-4-reductase* (*DFR*) was upregulated in the pathway that was a key enzyme regulating anthocyanin and proanthocyanin synthesis in the flavonoid biosynthesis pathway (Chen et al., 2020). In EF vs. LF, “starch and sucrose metabolism” was the mainly enriched pathway. The two pathways were also reported in the integrative analysis of flower bud differentiation, flower bud elongation, and floral anthesis in Loquat (*Eriobotrya japonica*) (Jing et al., 2020). Based on these KEGG pathways, not only known flowering genes but also transcription factors, and plant hormone signal transduction, were selected as the flowering candidate genes of *E. sibiricus*. Most genes from MYB, bHLH, AP2, WRKY, and MADS families have positive regulation on flower transformation (Matías-Hernández et al., 2016). *WRKY75*, a WRKY DNA-binding protein, positively regulates flowering by acting on *FT*, and thus, overexpression of *WRKY75* can promote flowering in Arabidopsis (Zhang et al., 2018b). *REM16* is one of the members of AP2/B3-like transcription factor family that acts upstream of *SOC1* and *FT* in the flowering pathway and delays the flowering when it silences (Yu et al., 2020). Previous studies showed that transcription factors of NAC, bZIP, and Homebox families accelerated the flowering and enhanced plant tolerance to drought stress (Jakoby et al., 2002; Minh-Thu et al., 2018; Zhang et al., 2018a). Heat shock transcription factors (HSFs) are involved in flowering development and heat stress response by activating heat shock proteins (Liang et al., 2021). It is concluded that some transcription factors are not only involved in flowering development but also respond to abiotic stress, which is beneficial to the cultivation of early- and late-flowering-resistant varieties.

Plant hormone signaling coordinates flowering in higher plants. We found candidate genes related to auxin, gibberellins (GAs), abscisic acid (ABA), salicylic acid (SA), and jasmonic acid (JA) involved in *E. sibiricus* flowering process. Auxin, GAs, and ABA are the traditional phytohormones. The most common auxin is indole-3-acetic acid (IAA). IAA and GA regulate DELLA protein which inhibits flowering, such as *AUXIN RESPONSE FACTOR* (*ARF*) and *GA-insensitive* (*GAI*) (Yamaguchi et al., 2013). The orthologs of *ARF* were also found in our study. Abscisic acid receptors *PYL4, PYL5*, and *PYL9* were differentially expressed in the early- and late-flowering accessions of *E. sibiricus* in our study. A recent study showed that *ABSCISIC ACID-INSENSITIVE MUTANT* 5 (*ABI5*) modulated the *PYL*-mediated ABA responses to promote flowering by activating *FT* (Zhao et al., 2020a). SA and JA are the plant hormones that are attaining deep concerns in recent years because they are actively involved in responses to stress conditions in plants. *Salicylic acid-binding protein 2* (*SABP2*) found in this study was only reported to be related to stress resistance (Haq et al., 2020), whereas *JASMONATE-RESISTANT1* (*JAR1*) was involved in the photoperiod pathway (Chen et al., 2018). Previous studies have revealed that SA inhibited flowering through *SUMO E3 ligase* (*SIZ1*) which facilitates protein conjugation with a small ubiquitin-like modifier of *FLD*, and the action of *CORONATINE INSENSITIVE 1* (*COI1*) can be inhibited by JA (Campos-Rivero et al., 2017). The hormone regulation of flowering time in *E. sibiricus* should be further explored. The putative flowering regulation network constructed in the study has a reference value for flowering regulation of *E. sibiricus*.

### Flowering Hub Genes Selected by WGCNA

A total of seven hub genes were found in the blue module and the turquoise module that could be the key genes in future research on *E. sibiricus* flowering regulation. The genes *LIMYB, PEX19*, and *GWD3* may be the flowering promoters of *E. sibiricus*. *LIMYB* is an MYB domain protein that interacts with L10 and belongs to the MYB transcription factor family, which is widely involved in plant growth and development and metabolic regulation (Zorzatto et al., 2015). Many transcription factors from the MYB family are involved in flowering regulation, such as *EARLY FLOWERING MYB PROTEIN* (*EFM*), which directly inhibited *FT* expression in leaf vasculature and delayed flowering time in *A. thaliana*, so it can be speculated that *LIMYB* gene may be involved in the regulation of flowering time of *E. sibiricus* (Yan et al., 2014). The peroxisome is essential for normal development in plants. In Arabidopsis, *PEX19* plays an important role in the biosynthesis and transport of peroxisome membrane proteins, and peroxisome division and inheritance (McDonnell et al., 2016). It is closely related to cell senescence and may be involved in flowering regulation through the age pathway. Glucan water dikinase (GWD) enzymes are the catalytic enzymes in plant starch phosphorylation and play key roles in starch metabolism; among them, *GWD3* is one of the two clades of the GWD isoforms (Mdodana et al., 2019). It could affect grain filling, starch accumulation, and starch biosynthesis of *E. sibiricus* after flowering.

The genes *BOR7, PMEI28, LRR, and AIRP2* may be the flowering suppressors of *E. sibiricus*. *BOR7* is a boron transporter that carries an indispensable trace element during the flowering of plants. In *O. sativa*, the boron transporter, *OsBOR4*, is involved in normal pollen germination and tube elongation (Tanaka et al., 2013). It can also be verified whether *BOR7* affects pollen activity of *E. sibiricus* or not. *PMEI28* belongs to the carbohydrate esterase family. Pectin methylesterase can cut the ester bond between galacturonic acid and methyl, so the change in methyl esterification level plays a vital role in the growth and development of plants. Research has shown that overexpression of *OsPMEI28* in rice induced dwarf phenotypes and reduced stem diameter (Nguyen et al., 2017). *LRR* is the largest known subfamily of receptor-like protein kinases, which involves the whole life of plants, such as regulating plant growth and participating in stress response and defense response (Chakraborty et al., 2019). Similarly, the wheat *LRK10* gene involved in drought resistance was found to be related to the early flowering of alfalfa by RNA-seq (Ma et al., 2021). E3 ubiquitin ligase *AIRP2* could take part in the photoperiod pathway. Various components involved in the regulation of flowering photoperiod are the target proteins of E3 ubiquitin ligase, which can achieve ubiquitin degradation under the mediation of E3 ubiquitin ligase, resulting in photoperiod signal transformation and influencing the flowering of plants (Piñeiro and Jarillo, 2013). Moreover, *AtAIRP2* positively regulates abscisic acid (ABA) response in *A. thaliana* (Oh et al., 2017).

### Allelic Variation Analysis of Hub Gene *LIMYB*

The *L10-INTERACTING MYB DOMAIN-CONTAINING PROTEIN* (*LIMYB*) is a newly identified MYB family protein. The gene interaction with *NIK1* can completely downregulate the genes involved in the translation mechanism, and overexpression of *LIMYB* inhibits ribosomal protein genes at the transcriptional level, leading to inhibition of protein synthesis. At present, few works of literature have reported the role of *LIMYB* in plants. In this study, a single-nucleotide polymorphism (SNP) was found in the 320-bp ORF region of the hub gene *LIMYB* by allelic variation analysis which was not found in dbSNP (https://www.ncbi.nlm.nih.gov/SNP/). However, it is unclear that what types of biological functions are carried out through this SNP locus in *E.sibiricus*. There are two classical methods for SNP function verification. One is gene knockout which may not obtain phenotypes (Song et al., 2018; Wang et al., 2021), and the other is to predict changes in functional pathways through bioinformatic analysis and then conduct experimental verification (Ramsey et al., 2020). High cost and long test period are the difficulties of SNP function verification. Genome-wide association studies (GWAS) have become one of the most important methods for the identification of target quantitative trait loci (Paudel et al., 2021). Based on the GWAS results in soybean (*Glycine max*), two significant and sixteen non-significant SNP markers mapped to 18 candidate genes were involved in six major flowering pathways (Kim et al., 2020). In *O. sativa*, 309 SNPs related to flowering traits were identified by GWAS (Liu et al., 2021). In our subsequent experiment, GWAS will be conducted to verify whether the same SNP can be found in *LIMYB*. If so, functional validation will be conducted. Recently, reports have identified SNP characteristics that predict validation success in GWAS hits (Liu et al., 2021). These characteristics can be used to select SNPs for validation and downstream functional studies to provide the reference for our study.

### Expression Analysis of Flowering Candidate Genes Under Abiotic Stress

Many types of abiotic stresses usually induce flowering to ensure the survival of future generations, including light intensity, ultraviolet light, high temperature, low temperature, malnutrition, nitrogen deficiency, drought, salt, hypoxia, and so on (Wada and Takeno, 2010). Nevertheless, different stress factors can also inhibit or delay flowering in many plant species (Takeno, 2016). In this study, the expression pattern of flowering candidate genes between early- and late-flowering accessions in *E. sibiricus* was verified by qRT-PCR, containing eight accelerated flowering genes (*CRY, COL, FPF1, Hd3, GID1, FLK, VIN3*, and *FPA*) (Kania et al., 1997; Schomburg et al., 2001; Monna et al., 2002; Griffiths et al., 2003; Hirano et al., 2008; Liu et al., 2008; Kim et al., 2010; Rodríguez-Cazorla et al., 2015) and four delayed flowering genes (*CCA1, ELF3, Ghd7*, and *COL4*) (Xue et al., 2008; Lu et al., 2012; Steinbach, 2019; Zhao et al., 2021).

Under salt stress, the majority of candidate genes were upregulated in early-flowering accessions that resulted in acceleration or delay in flowering. In late-flowering accessions, all the genes promoting flowering were upregulated, and all the genes inhibiting flowering except *ELF3* were only expressed at 150 mmol/L NaCl treatment. The studies of salt stress originated from salt accumulation caused by irrigation. Previous report showed that the salt overly sensitive pathway and photoperiod pathway co-regulated flowering time and stress tolerance (Park et al., 2016). Both acceleration and delay in flowering are the defensive behaviors of plants under salt stress to ensure reproduction. But, a long delay in flowering under high concentration stress may lead to an insufficient reproductive time of late-flowering accessions and thus affect seed setting. Reports indicated that the expression of *ELF3* not only delayed flowering but also enhanced salt tolerance of soybean and Arabidopsis (Sakuraba et al., 2017; Cheng et al., 2020). The study also confirmed the function of the *ELF3* gene in *E. sibiricus*. Under drought stress, many plants speed up the flowering process in the absence of water to shorten their life cycle and this process is known as drought-escape (DE) response (Kooyers, 2015). In this study, the majority of candidate genes were upregulated under low concentration PEG6000 in both early- and late-flowering accessions. However, at 20% PEG6000 treatment, all genes that inhibit flowering were downregulated, showing a DE response. Furthermore, some plants have other strategies, such as *RICE CENTRORADIALIS 1* (*RCN1*), which delays the flowering of rice under drought stress (Wang et al., 2020). The flowering promoter *COL4* was found always downregulated under any concentration of drought in late-flowering accessions as presented in this study.

Overall, stress activated the flowering regulation pathway to some extent, and flowering candidate genes of *E. sibiricus* were expressed under drought and salt stress. Under high salt and drought stress, plants preferentially accelerate their life processes and produce seeds before death. The network between flowering genes and abiotic stress needs further investigations in *E. sibiricus* for the development of stress-tolerant varieties with high seed yield and biomass.

## Conclusion

In this study, we revealed flowering time variations of 66 *E. sibiricus* accessions collected from different countries and identified flowering regulatory networks and hub genes expressed in the leaves by comparative transcriptome analysis. A total of 3,526 DEGs were predicted and 72 candidate genes were identified, of which *LATE, GA2OX6, FAR3*, and *MFT1* were recognized as late-flowering genes. The WGCNA analysis revealed 7 hub genes controlling flowering in *E. sibiricus*. The result of multiple sequence alignment indicated that single-nucleotide polymorphism (SNP) of hub gene *LIMYB* may cause late flowering. The validation of the expression pattern of flowering candidate genes suggested that stress may activate the flowering regulation pathway to some extent. The research on flowering regulatory networks and these potential candidate genes will be valuable resources for further flowering functional research in *E. sibiricus*.

## Data Availability Statement

The *E. sibiricus* transcriptome sequencing data have been deposited in National Center for Biotechnology Information (NCBI), under accession number PRJNA665941.

## Author Contributions

WX conceived and designed the experiments and acquired financial support for the project leading to this publication. NW performed field management and sampling. YZ and NW performed the qRT-PCR and multiple sequence alignment experiments. YZ, NW, ZZ, and WL analyzed the data. YZ performed data visualization and wrote the initial draft. WX and YZ revised the manuscript. All authors have read and agreed to the published version of the manuscript.

## Funding

This research was funded by the Gansu Provincial Science and Technology Major Projects (19ZD2NA002), Chinese National Natural Science Foundation (31971751), the State's Key Project of Research and Development Plan (2019YFC0507702), the open projects of Key Laboratory of Superior Forage Germplasm in the Qinghai-Tibetan Plateau (2020-ZJ-Y03), and the Fundamental Research Fund for the Central Universities (lzujbky-2021-ct21).

## Conflict of Interest

The authors declare that the research was conducted in the absence of any commercial or financial relationships that could be construed as a potential conflict of interest.

## Publisher's Note

All claims expressed in this article are solely those of the authors and do not necessarily represent those of their affiliated organizations, or those of the publisher, the editors and the reviewers. Any product that may be evaluated in this article, or claim that may be made by its manufacturer, is not guaranteed or endorsed by the publisher.

## Supplementary Material

Supplementary Figure 1 The clustering heat map of samples for transcriptome analysis.

Supplementary Figure 2 The unigenes distribution of *E. siburicus*.

Supplementary Figure 3 The top 10 species distribution of transcripts that were annotated on the basis of homology.

Supplementary Figure 4 KEGG classification map **(A–C)** and enrich scatter plot of KEGG pathway **(D–F)** of DEGs. **(A,D)** EB vs. LB. **(B,E)** EH vs. LH. **(C,F)** EF vs. LF.

Supplementary Figure 5 The bar chart of correlation between DT trait and module genes.

Supplementary Table 1 The origin of 66 *E. siburicus* accessions.

Supplementary Table 2 The primers information of *E. siburicus*.

Supplementary Table 3 Sequencing data evaluation statistics in *E. sibiricus*.

Supplementary Table 4 The statistics of unigenes annotation in *E. sibiricus*.

Supplementary Table 5 The information of flowering pathway genes.
