# Supplementary figures and images for: Identification of Flowering Regulatory Networks and Hub Genes Expressed in the Leaves of Elymus sibiricus L. Using Comparative Transcriptome Analysis
Source: Front Plant Sci. 2022 May 16;13:877908. doi: 10.3389/fpls.2022.877908 (PMC9150504; doi:10.3389/fpls.2022.877908)

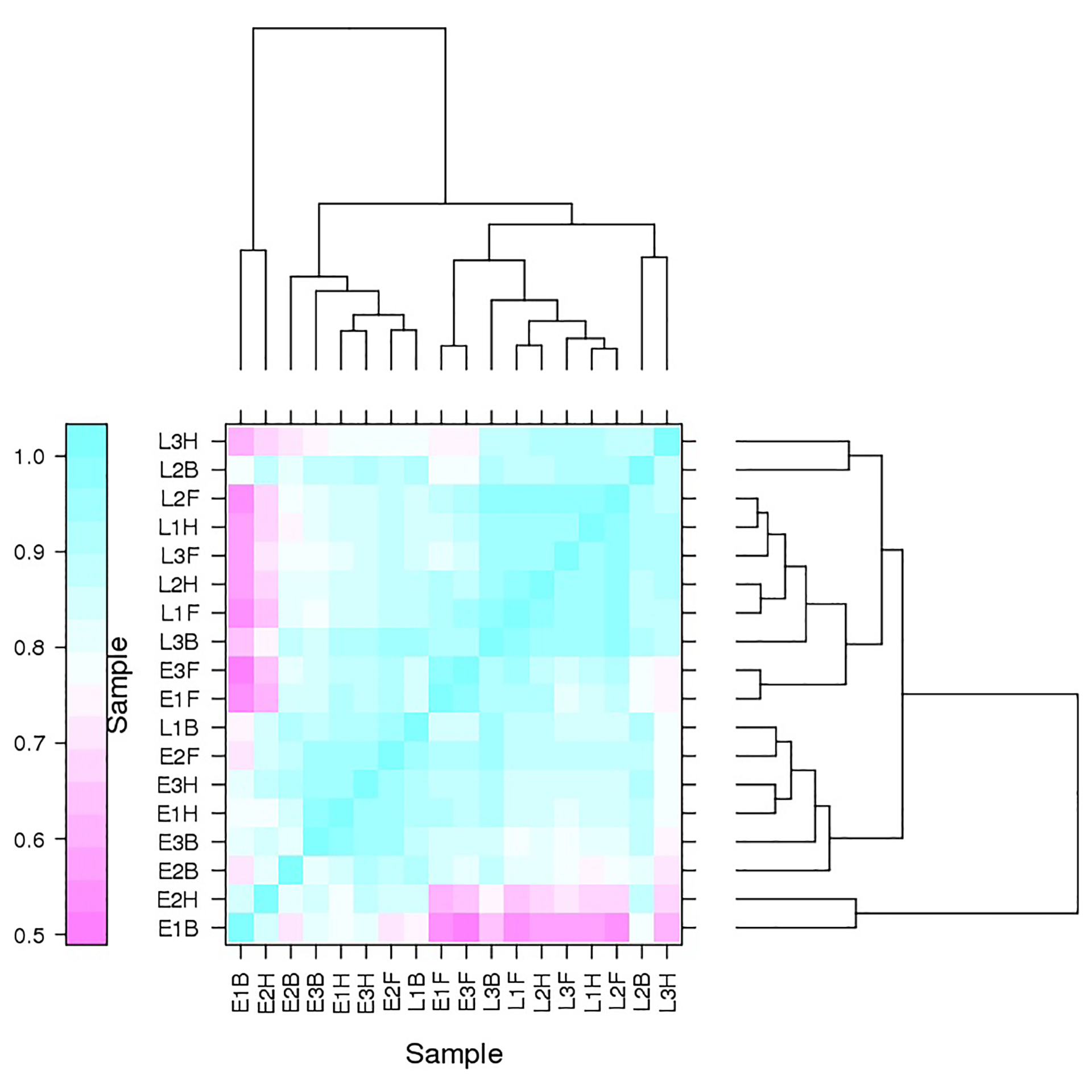

Supplement: Supplementary Figure 1 — The clustering heat map of samples for transcriptome analysis. [file Image_1.JPEG]

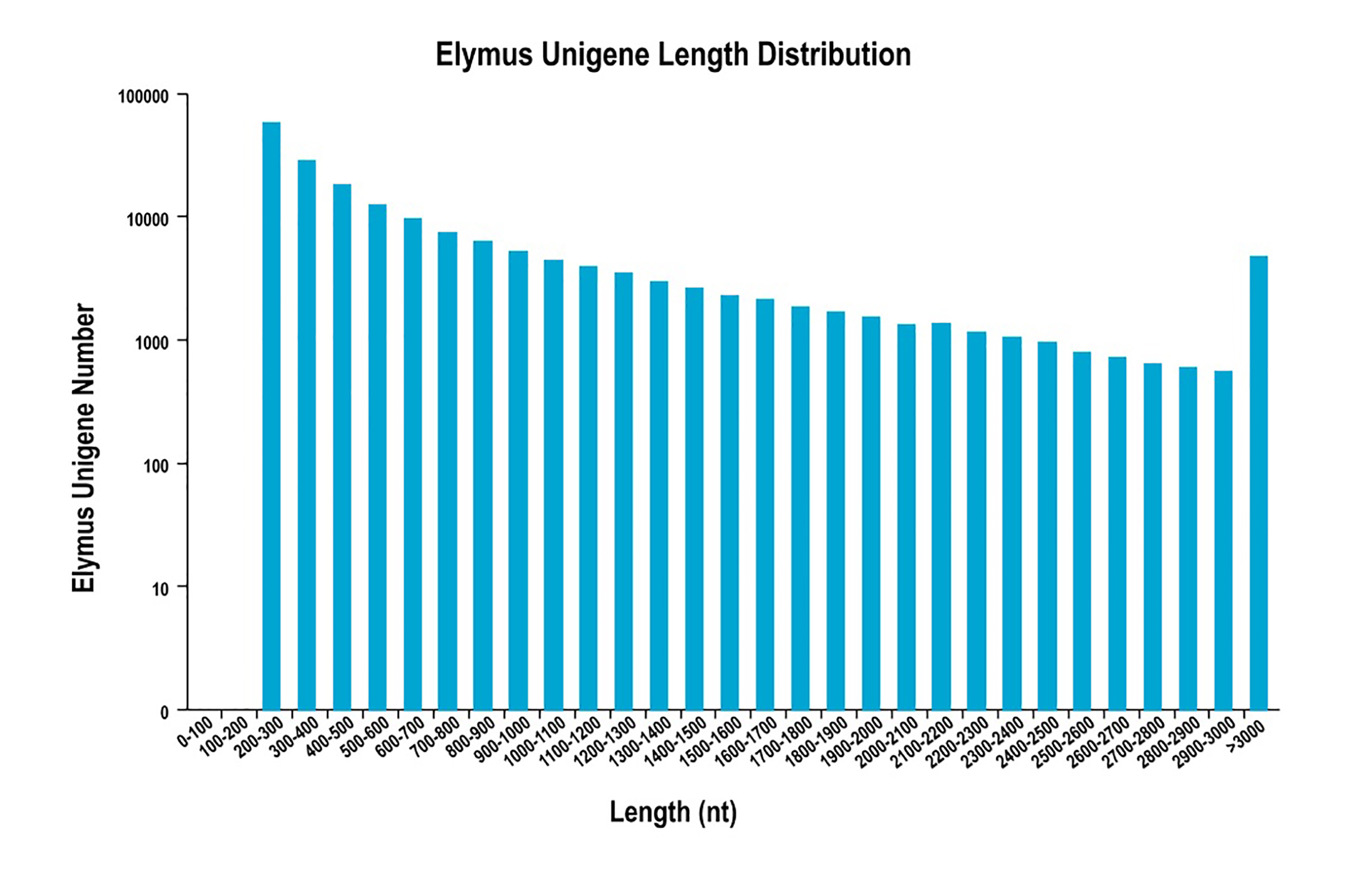

Supplement: Supplementary Figure 2 — The unigenes distribution of E. siburicus. [file Image_2.JPEG]

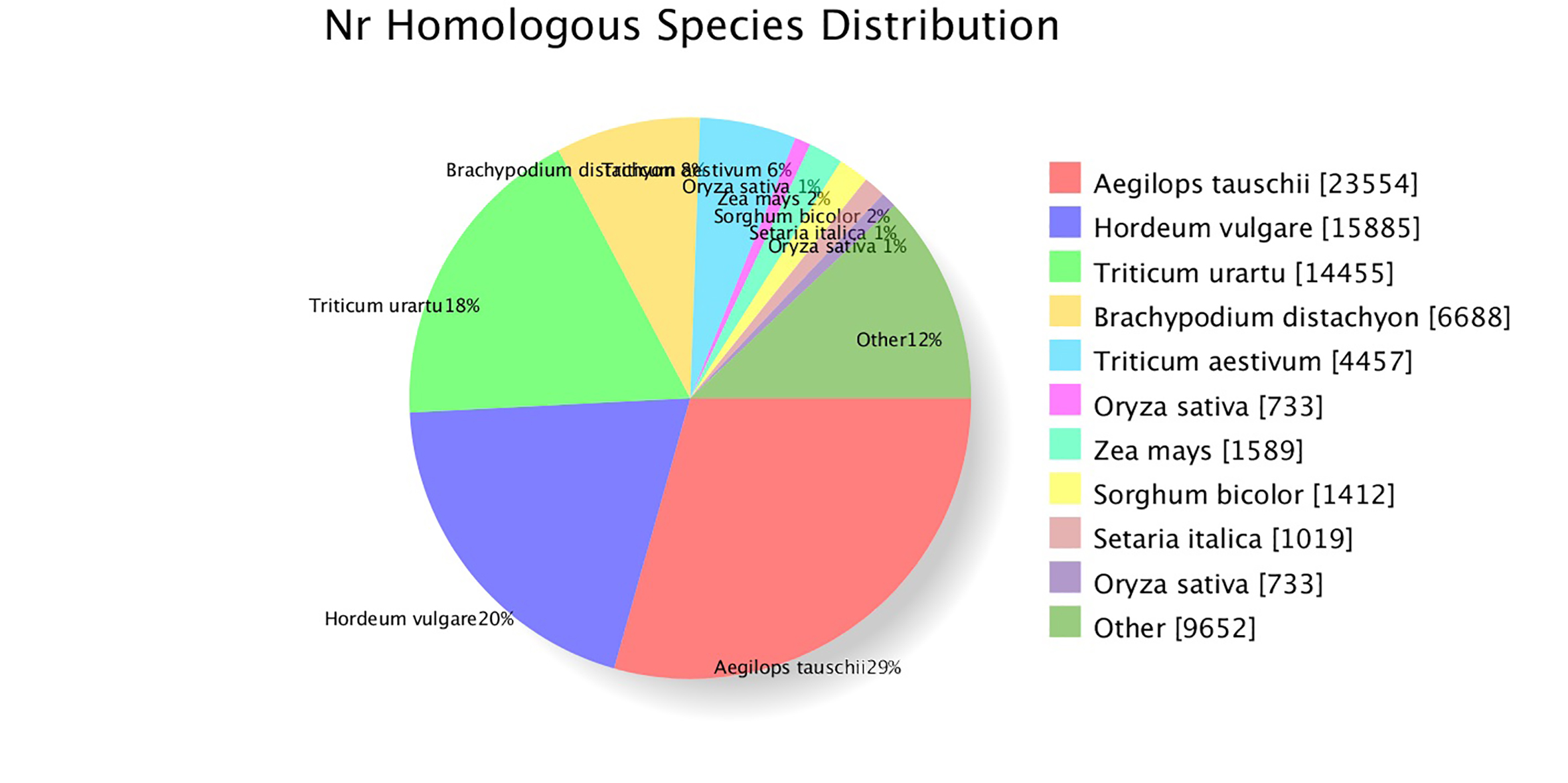

Supplement: Supplementary Figure 3 — The top 10 species distribution of transcripts that were annotated on the basis of homology. [file Image_3.JPEG]

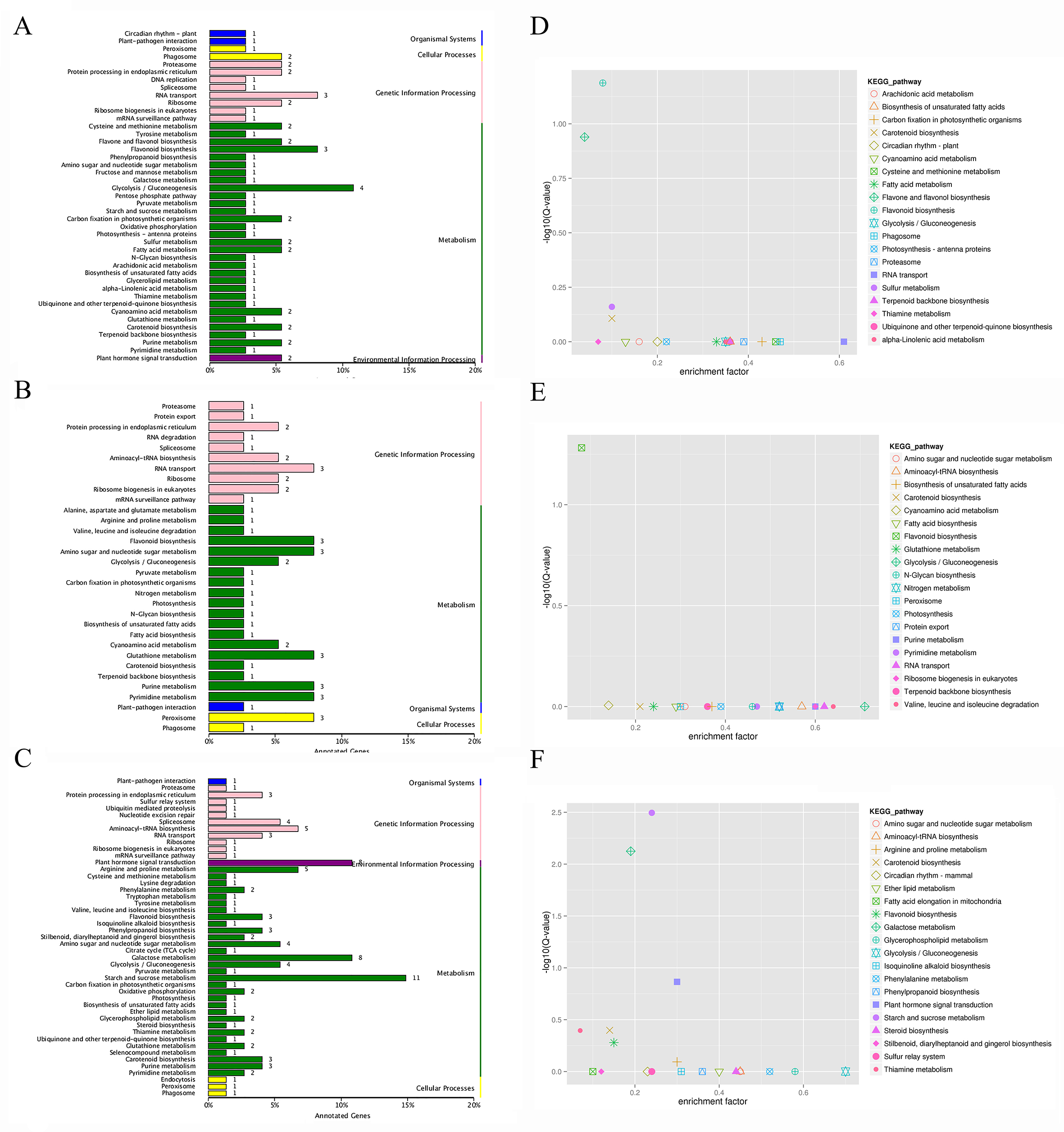

Supplement: Supplementary Figure 4 — KEGG classification map (A–C) and enrich scatter plot of KEGG pathway (D–F) of DEGs. (A,D) EB vs. LB. (B,E) EH vs. LH. (C,F) EF vs. LF. [file Image_4.JPEG]

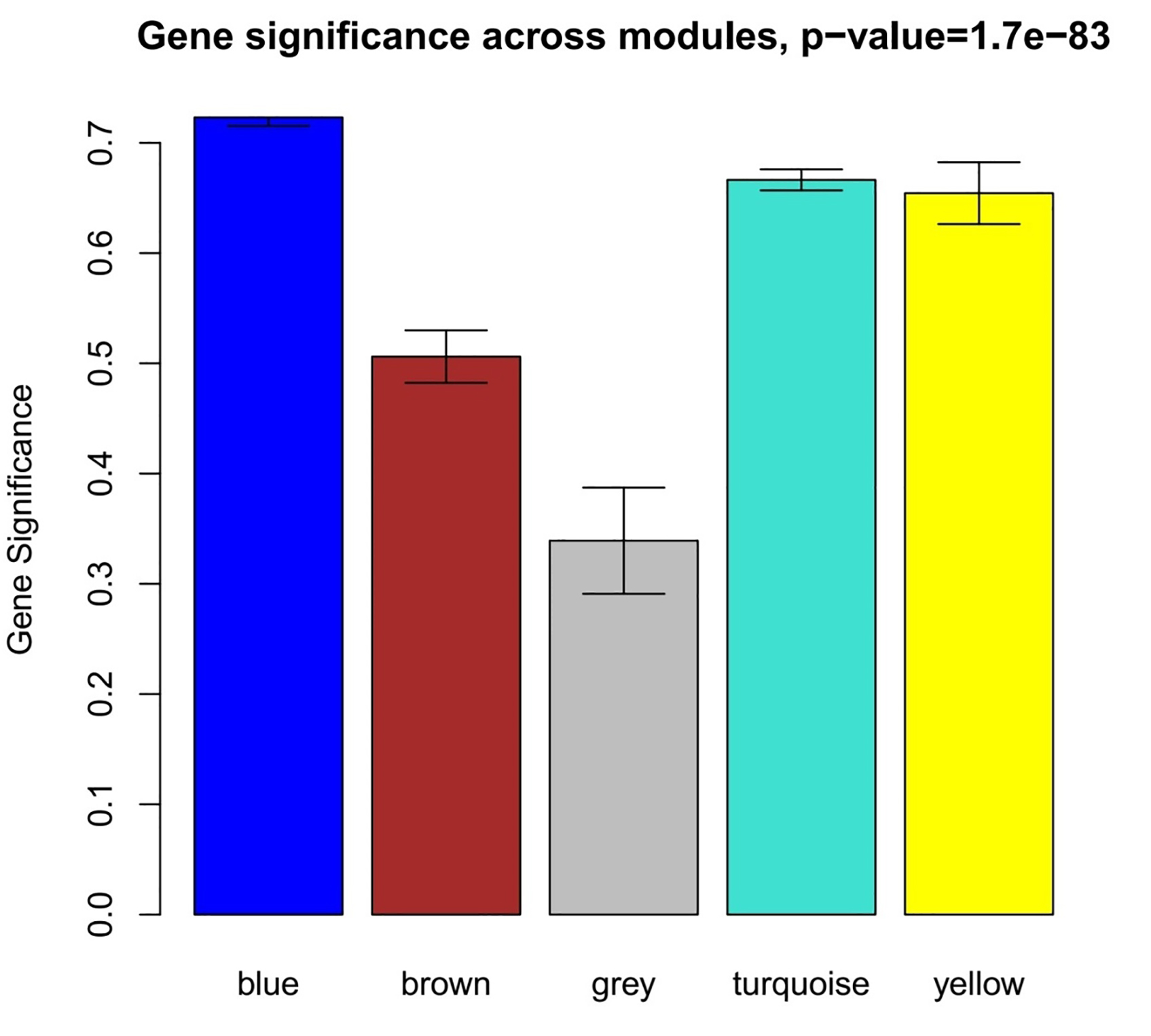

Supplement: Supplementary Figure 5 — The bar chart of correlation between DT trait and module genes. [file Image_5.JPEG]
